# Genetic relatedness reveals total population size of white sharks in eastern Australia and New Zealand

**DOI:** 10.1038/s41598-018-20593-w

**Published:** 2018-02-08

**Authors:** R. M. Hillary, M. V. Bravington, T. A. Patterson, P. Grewe, R. Bradford, P. Feutry, R. Gunasekera, V. Peddemors, J. Werry, M. P. Francis, C. A. J. Duffy, B. D. Bruce

**Affiliations:** 1 CSIRO Oceans and Atmosphere GPO Box 1538, Hobart, TAS 7000 Australia; 2 CSIRO Data61, GPO Box 1538, Hobart, TAS 7000 Australia; 3 New South Wales Department of Primary Industries, Sydney Institute of Marine Science 19 Chowder Bay Road, Mosman, NSW 2088 Australia; 4 Griffith Centre for Coastal Management, Griffith University, Southport, QLD 4226 Australia; 5 National Institute of Water and Atmospheric Research, Private Bag 14901, Wellington, 6022 New Zealand; 6 Department of Conservation, Private Bag 68908, Newton, Auckland, 1145 New Zealand

## Abstract

Conservation concerns exist for many sharks but robust estimates of abundance are often lacking. Improving population status is a performance measure for species under conservation or recovery plans, yet the lack of data permitting estimation of population size means the efficacy of management actions can be difficult to assess, and achieving the goal of removing species from conservation listing challenging. For potentially dangerous species, like the white shark, balancing conservation and public safety demands is politically and socially complex, often leading to vigorous debate about their population status. This increases the need for robust information to inform policy decisions. We developed a novel method for estimating the total abundance of white sharks in eastern Australia and New Zealand using the genetic-relatedness of juveniles and applying a close-kin mark-recapture framework and demographic model. Estimated numbers of adults are small (ca. 280–650), as is total population size (ca. 2,500–6,750). However, estimates of survival probability are high for adults (over 90%), and fairly high for juveniles (around 73%). This represents the first direct estimate of total white shark abundance and survival calculated from data across both the spatial and temporal life-history of the animal and provides a pathway to estimate population trend.

## Introduction

Top-order predators retain a very visible presence in human society due to their size, power, dramatic interactions with prey and infrequent, but high profile, interactions with humans that sometimes result in tragic outcomes. The latter generates considerable public and political debate, particularly for protected species, requiring a delicate balance between maintaining public safety and population recovery^1^. The white shark (*Carcharodon carcharias*) is emblematic of this duality. It is globally distributed, long lived (>50 yrs), attains up to 6.5 m, and has low reproductive potential making populations vulnerable to decline from human impacts^2,3^. It has gained notoriety from attacks on humans and through its prominence in popular culture^4^. White sharks are listed under international conventions restricting global trade and coordinating conservation measures. They are listed as *Vulnerable* by the International Union for the Conservation of Nature (IUCN), on Appendix II of the Convention on International Trade in Endangered Species (CITES) and on both Appendices II and III of the Convention on the Conservation of Migratory Species (CMS)^5^. They are protected in the national waters of several countries due to documented or perceived population declines and vulnerabilities given its life history^2^. White sharks are protected in Australia, listed as both *Vulnerable* and *Migratory* under the Federal Environment Protection and Biodiversity Conservation Act 1999, protected under various State legislation and subject to a national recovery plan to arrest decline and improve population status^6^. Despite global progress on identifying movement patterns, habitat and population structure, there is still a poor understanding of the size and status of white shark populations across their range, including Australia^7^. This is due to the lack of data useful for reconstructing population levels and trends, a common problem for many shark species of conservation concern^5^.

Attempts to estimate abundance of white sharks have previously focused sampling effort at known aggregation sites. Studies have used individual photographic records, reconstructed patterns of annual return and tag-recapture models to estimate the number of sharks visiting these sites^8,9^. However, the accuracy of photo-identification techniques and the population models used by these studies suffer from several biases which can be difficult to account for^9,10^. Some studies have extrapolated results to provide regional population estimates^11,12^, but extrapolating site-specific estimates to regional levels can be compromised by sharks’ site fidelity, life-history-specific habitat use and factors impacting the probability of recording and re-sighting an individual^9,10,13^. Two studies have attempted population estimates based on conventional tag-recapture^14,15^, but these have similar challenges in addition to issues around tag loss and low sample sizes. Establishing current status and trends in marine populations generally requires long-term, standardized, historical records or other suitable abundance indices. However, these are often not available. Historical catches of white sharks from commercial or recreational fisheries conceptually offer some promise, but changing patterns of spatial effort, poor and inconsistent records, as well as management changes which impact catchability, generally render such data inadequate^16^. While such analyses provide indications of decline in the western Atlantic^17^, and possible juvenile increase in Californian waters^18^, establishing total population size and hence, an understanding of population status in white sharks, has hitherto proved elusive. Recent public and political debate in response to fatal attacks by white sharks^19^ has highlighted an urgent need to assess their population status. This is required to establish the efficacy of conservation actions, design effective and defensible population rebuilding strategies, and to provide a scientifically sound and rational basis from which to develop policies that balance conservation objectives and public safety. To circumvent these difficulties, we use a novel approach which relies on some features of regional white shark biology^2,7^. We summarise the relevant background information next and then outline the method.

White sharks in Eastern Australia and New Zealand form a single population, which was exposed to appreciable human-induced mortality in the middle of the 20th century, but has been protected since the mid-1990s. Individual adults and sub-adults range widely across the region, whereas juveniles keep to a more coastal distribution, and some age classes congregate seasonally along the coast of the state of New South Wales. Females reach maturity around age 15, and at a large size (close to 5 m) which is close to their maximum size; males are somewhat smaller. Litter sizes are variable but modest (6–7 would be typical), and there is no indication that litter size is related to female body size. Inter-birth interval is at least one year and adults can certainly live for several decades, although maximum lifespan is a subject of controversy^3^. At least for immature white sharks, length is a useful albeit variable predictor of age (see Section S2.4 of the supplementary material). Additionally, the observed growth increments of tagged juvenile white sharks that are subsequently recaptured - which are independent of the ageing method - match closely with the predictions from the length-at-age relationship. Since we only requires age estimates for juveniles, our results are not susceptible to possible underestimation of adult age from vertebral rings, which has been demonstrated for some elasmobranch species^3^.

Adult white sharks in Eastern Australia and New Zealand cannot be sampled in useful numbers, but genetic sampling of juveniles is possible both through live-release biopsies at a congregation site, and through lethal capture in shark control nets. Juveniles can also have long-term acoustic tags implanted, which can be detected in subsequent years across a network of acoustic receivers along the east Australian coast; this provides information on juvenile survival. Our overall approach is as follows:

- genotype juvenile samples thoroughly enough to identify Half-Sibling Pairs (HSPs), i.e. which share one parent;
- from the proportions of HSPs to Unrelated Pairs (UPs), estimate adult abundance, trend, and survival rate;
- from acoustic tag data, estimate juvenile survival rate using a mark-recapture model;
- from all estimated parameters, and other vital rate information, infer the abundance of juveniles required to balance the demographics, using a Bayesian Leslie-matrix model.

The second step uses the recently-developed technique of Close-Kin Mark-Recapture (CKMR)^20^, a novel extension of traditional mark-recapture approaches^21^ first applied in a parent-offspring context to estimate the spawning population of southern bluefin tuna (*Thunnus maccoyii)*^20,22^. The key linkage to mark-recapture approaches is that each juvenile carries the “marks” of its parents within its DNA, and to “recapture” these marks we use modern genetics to find juveniles who share a common parent. To outline the general concepts, consider two juveniles born a few years apart, and suppose that their ages (i.e. their years-of-birth) are known. Assuming that the mother of the first-born is equally like to have been any of the *N*_♀_ females, the probability that the second animal’s mother happens to be the same animal is intuitively about 1/*N*_♀_. However, if the gap between the years-of-birth is long enough, the first animal’s mother might have died before the second animal was born, which reduces the probability; and the probability will also be affected by variations in the overall number of females over time. These same ideas extend to the father-offspring case and, hence, to total adult population. Thus, the observed proportions of HSPs to UPs (taking into account juvenile birth-years) carry information on the level and trend of adult abundance, and on adult survival. The Methods section explains how we accommodated various important details, such as same-cohort comparisons, and how the various analyses (for both juveniles and adults) are integrated to obtain total population abundance estimates.

## Results

### Finding half-sibling pairs among the juvenile samples

**Figure 1:**
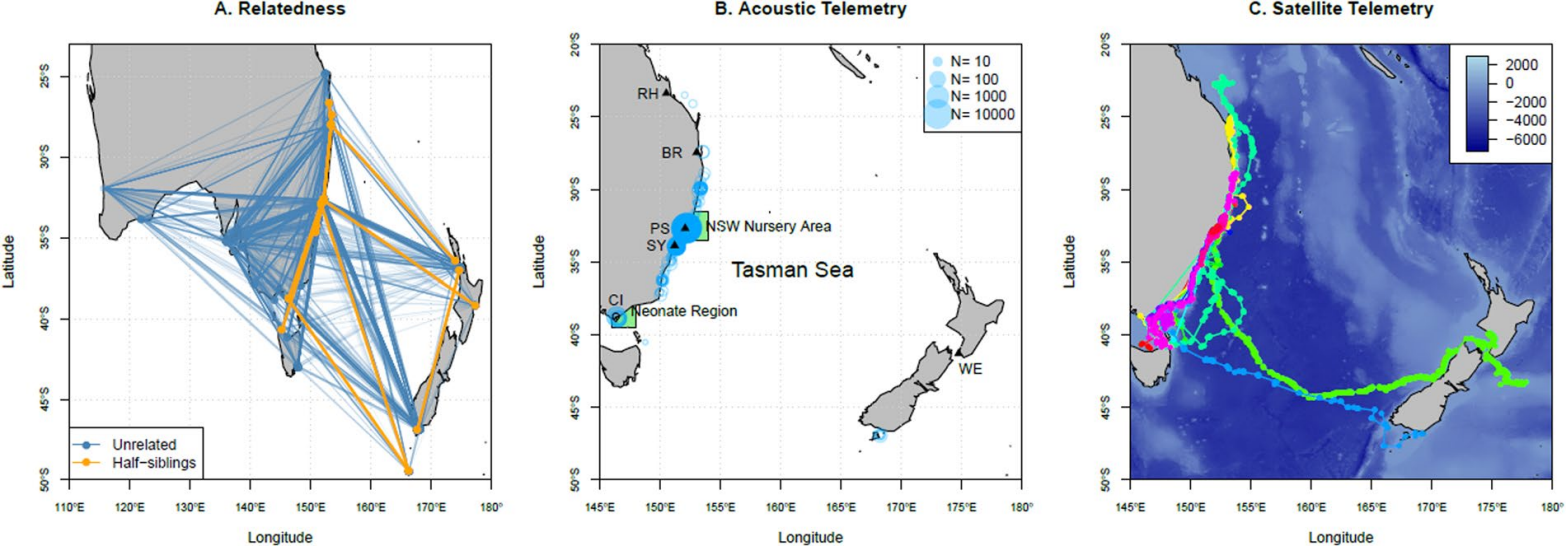
Data collection. (**A**) Relatedness: Pairwise comparisons between juvenile white sharks (JWS) collected at specific locations shown linked by lines. Blue lines indicate comparisons where sharks were unrelated; orange lines identify half-siblings (HSPs). HSPs were geographically restricted to eastern Australia and New Zealand supporting the two population model for Australasia. HSPs were mixed throughout the range of the sample population indicating a lack of bias in the opportunistic sampling strategy. (**B**) Acoustic Telemetry: Distribution of acoustic detections of tagged JWS. Green boxes indicate the location of nursey areas where sharks were tagged. (**C**) Satellite Telemetry: Satellite tracks of tagged JWS tagged in NSW and Vic nursery areas. Both satellite tracking and acoustic telemetry data are consistent with the known distribution of white sharks in eastern Australia. RH = Rockhampton, BR = Brisbane, PS = Port Stephens, SY = Sydney, CI = Corner Inlet, WE = Wellington. All map figures were generated using the R^39^ packages ggplot2^40^, ggmap^41^, and gridExtra^42^ found on the Comprehensive R Archive Network (https://cran.r-project.org/).

We genotyped 183 white sharks collected from across their Australasian range, including New Zealand, at 8,961 loci (Fig. 1A). A strict quality control procedure was followed to check all the relevant genetic aspects of the data, and is explained in detail in Sections S2.1 through S2.3 of the supplementary material. First, the statistical consistency of the loci and samples was checked against the allele frequency model, ensuring the statistical model describing the allele frequencies was able to represent those appearing in the sampled animals. Loci or samples that failed were excluded from further analyses, thereby removing potentially contaminated samples and avoiding atypical loci as both could introduce biases. This reduced the sample size to 115 sharks and 2,186 loci. The pattern of rejected samples under the quality control procedures suggested that these losses were probably due to sample contamination resulting in anomalous allele frequencies in these fish, relative to the rest of the sample. Removing these samples would not incur a bias, it simply reduces the level of precision we can expect to obtain in the final estimates. Not removing these samples would almost certainly incur a degree of bias as these fish, whose samples seemingly contain amounts of each other’s DNA to some degree, will anomalously alter the number of detected half-siblings. Additionally, quality control procedures identified nine duplicate samples/repeat recaptures and four full-sibling pairs (juveniles sharing both parents). These were removed to avoid them aliasing as half-siblings. Two more samples lacked length information, leaving samples from 100 sharks. Of these, 75 were sampled in eastern Australia and New Zealand (herein ‘Eastern’) and 25 were sampled in western Victoria, South Australia or Western Australia (herein ‘Western’). These 100 samples provided 4,950 unique pair-wise genotype comparisons from which 20 half-sibling pairs (HSPs) (+/−1 false positive/negative) were found within the Eastern samples; one Western HSP was also detected (though another was found with one shark lacking the requisite length information and not included in the final 100 samples). No sharks from the Eastern samples were identified as HSPs with Western sharks, supporting previous evidence for separate populations in these regions^23^. Sharks identified as HSPs were spread across the geographic range of the Eastern population indicating a lack of site-specific bias in sampling (Fig. 1), akin to the requirement that marked and recaptured animals are ‘well-mixed’ in the population in regular mark-recapture model assumptions. Hence we focus on the 75 Eastern sharks (2,775 unique comparisons).

### Demographic modelling of adult population dynamics using half-sibling pairs

Whole mitogenome sequencing was completed for all 20 HSPs which identified 14 haplotypes. Twelve of these 20 HSPs shared a haplotype and eight did not. This observation was consistent with a 50:50 sex ratio in the adult population, and no strong reproductive skew within males with. In white sharks there is no evidence for significant growth post-maturation for females that would possibly lead to a change in fecundity with increasing maternal age or size^2^, and no biologically tenable rationale for assuming that male reproductive success is strongly linked to age. This suggests using a demographic model where all adults are considered equal in terms of expected reproductive output, and underpins our decision to assume an age and sex aggregated model for the adult population dynamics. Thus the population model consists of adult abundance, *N*^*A*^, (both males and females) over time with a constant survival probability, *ϕ*^*A*^, and (logarithmic) growth/decline rate, *λ*. Given this structure, the equation for the adult population over time is as follows:

1 $${N}_{t}^{A}={N}_{init}{e}^{\lambda t}$$

where *N*_*init*_ is the total adult abundance in the initial model year. The probability of two juveniles being half-siblings depends on adult abundance *and* survival in the time between the births of the two juveniles (see Methods section), so we require a third parameter *ϕ*^*A*^ for adult survival probability.

The cohort (i.e. birth year) of each juvenile is very important in calculating half-sibling probabilities. The cohort, *c*, of the animal is simply determined as the year *y* it was sampled in, minus its age *a* at sampling: *c* = *y* − *a*. The sampling year is known without error for all our animals, and length-at-sampling was known for all animals in the subset used in the adult demographic model. Where available, the ages of the animals were obtained based on band-pair counts in their vertebrae^24^. Where vertebral age estimates were not available- for example when sharks were sampled, tagged and released- the distribution of juvenile age-given-length was calculated and used to probabilistically account for uncertainty in age (and by implication, birth cohort) given length-at-sampling in the model (see supp. mat Section S2.4).

The number of HSPs and limited range of cohorts observed (relative to generation time) meant that the model provided clear information on abundance and survival, but was largely uninformative for adult population growth rate (*λ*). A combination of evidence, including a lack of any clear genetic bottleneck effects and biologically feasible maximum population growth rates given known life-history characteristics, was used to define scenarios ranging from population decline through increase for *λ* = 0, ± 0.02, ± 0.04 from which to estimate adult abundance (Supplement S2.7.3). Using the year 2000 as a reference, just after protection of white sharks in Australian waters in the mid to late 1990s, the *λ* scenarios result in adult abundance estimates in 2015 ranging from 54% to 182% of year 2000 levels.

**Figure 2:**
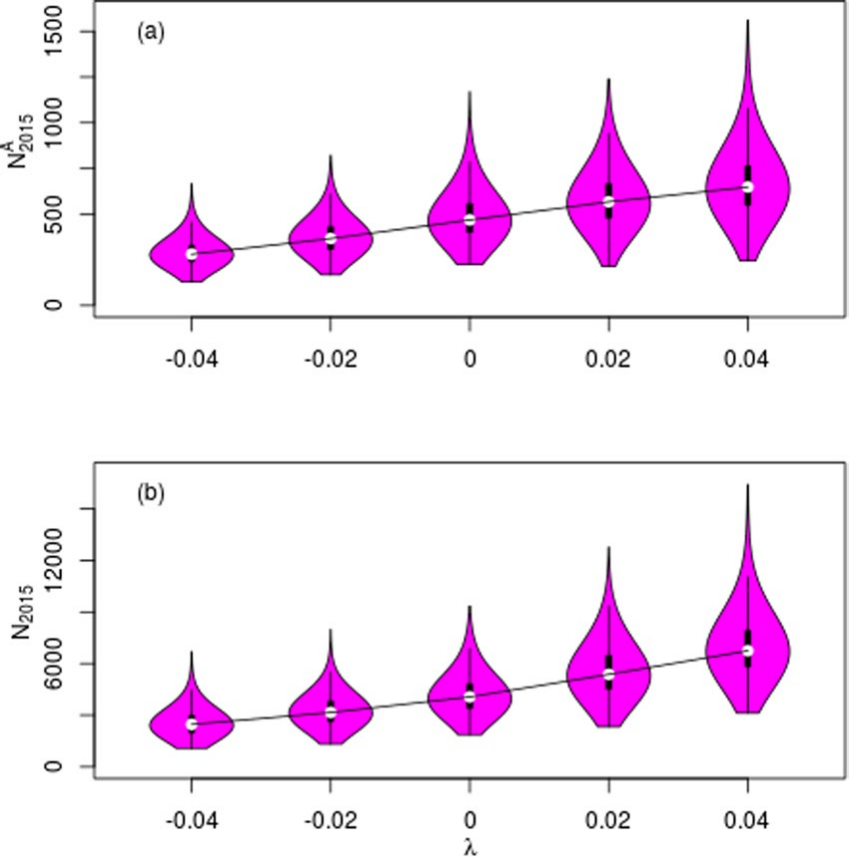
(**a**) and (**b**): Violin plots of (**a**) adult and (**b**) total abundance in 2015. The purple region reflects the probability distribution; the filled rectangle the inter-quartile range; the dotted box the 95% credible interval; the grey points the median and mean; and the solid black line the trend in the medians. The x-axis denotes the 5 population rate-of-change scenarios: *λ* = *0*, ± *0.02*, ± *0.04*.

Figure 2(a) summarises the close-kin derived adult abundance and survival estimates under these population growth scenarios (*λ*) (see also Table 1 supp. mat.). Adult survival estimates were high and reasonably consistent across scenarios, with medians ranging from 0.94 to 0.98 and 95% credible intervals between 0.9 and 0.99. While high, such values are not surprising given the ecological niche and longevity of such a top-order predator. Median (and 95% credible interval) estimates of current (as of 2015) adult abundance were: *N*^*A*^ = 280 (184–460) for *λ* = −0.04, *N*^*A*^ = 365 (226–783) for *λ* = −0.02, *N*^*A*^ = 467 (282–783) for *λ* = 0, *N*^*A*^ = 567 (340–923) for *λ* = 0.02 and *N*^*A*^ = 647 (392–1,096) for *λ* = 0.04.

**Table 1:** Summary (pre and post-ABC algorithm where relevant), in terms of median and 95% credible intervals, for total population abundance in 2015, survival rates (juvenile and adult and age-0 multiplier *δ*) and realised fecundity (*α*) for each of the 5 population growth scenarios.

Case *(λ)*	*N*_*2015*_	*ϕ* ^*J*^	*ϕ* ^*A*^	*α*	*δ*
Pre ABC (−0.04)	—	0.73 (0.62–0.81)	0.94 (0.89–0.95)	1.5 (1–2.5)	0.82 (0.53–0.97)
Post ABC (−0.04)	2,467 (1,464–4,381)	0.74 (0.72–0.78)	0.94 (0.92–0.95)	1.75 (1–2.5)	0.86 (0.62–0.98)
Pre ABC (−0.02)	—	0.73 (0.62–0.81)	0.96 (0.91–0.97)	1.5 (1–2.5)	0.82 (0.53–0.97)
Post ABC (−0.02)	3,161 (1,842–5,272)	0.75 (0.73–0.8)	0.96 (0.93–0.97)	2 (1.17–2.5)	0.86 (0.62–0.98)
Pre ABC (0)	—	0.73 (0.62–0.81)	0.98 (0.91–0.99)	1.5 (1–2.5)	0.82 (0.53–0.97)
Post ABC (0)	4,064 (2,451–7,020)	0.77 (0.75–0.82)	0.98 (0.93–0.99)	2 (1.33–2.5)	0.86 (0.63–0.98)
Pre ABC (0.02)	—	0.73 (0.62–0.81)	0.98 (0.91–0.99)	1.5 (1–2.5)	0.82 (0.53–0.97)
Post ABC (0.02)	5,375 (3,193–9,106)	0.81 (0.78–0.84)	0.98 (0.95–0.99)	2 (1.5–2.5)	0.88 (0.64–0.98)
Pre ABC (0.04)	—	0.73 (0.62–0.81)	0.98 (0.91–0.99)	1.5 (1–2.5)	0.82 (0.53–0.97)
Post ABC (0.04)	6,748 (4,138–11,083)	0.83 (0.82–0.87)	0.98 (0.96–0.99)	2 (1.5–2.5)	0.89 (0.72–0.98)

### Juvenile survival rate estimates

In addition to adult abundance estimates, estimation of total population size requires estimates of juvenile survival, with juveniles being animals between the ages of 0 to 10 in the acoustic data. Conventional mark-recapture experiments are impractical for this purpose in white sharks due to low recapture probabilities. Instead, we analysed detection histories from acoustic telemetry to infer juvenile survival. Forty-nine juvenile white sharks were fitted with internal acoustic tags along the East Australian coast^25^. Wide-scale monitoring of movements (and hence survival) was achieved via a network of acoustic receivers along the east Australian coast maintained by various organisations coordinated under Australia’s Integrated Marine Observing System (IMOS, see https://aatams.emii.org.au/aatams/installation/list?max = 20&offset = 0) and including receivers in southern New Zealand^26^. To determine the representativeness of the acoustic telemetry data, movements were also monitored in 32 of the same sharks via secondary tagging with fin-mounted satellite tags (see Section S1.2 and Tables S2, S3 and S4 in the supplementary material for a detailed release and detection summary)^25^. Tagging of juveniles yielded 186,743 individual detections on 300 receivers over a 7 year period (Fig. 1B). Detection records extended along the Australian coast from Tasmania to Queensland and across the Tasman Sea to New Zealand covering the full range of movements determined by the concurrent satellite tracking (Fig. 1C). Assuming constant, age-independent juvenile survival (*ϕ*^*J*^) we estimated *ϕ*^*J*^ = 0.73 (95% CI 0.62–0.81).

### Total population size and demography estimates

A combination of life-history theory and Approximate Bayesian Computation (ABC) was used to obtain estimates of the total population^27^. Given the imprecise estimates of population growth rate from CKMR, we continued to explore the five scenarios for possible growth rate considered in the CKMR analyses. A Leslie matrix model^28^ was constructed for the whole population, using available information on maturity and fecundity levels^29,30^ and our estimates of juvenile and adult survival. In principle, a fully time and age-dependent model could be developed to integrate all the available data. The reasons for not taking this approach were: (i) there was insufficient information in the current close-kin data to warrant building a more complex model; and (ii) by using a quasi-equilibrium (i.e. stable age composition) approach via the Leslie matrix model, we could accommodate the uncertainty in all the estimates in a probabilistically coherent fashion. Further, this framework clearly demonstrates how alternative population growth rates impacted the abundance and survival estimates.

The ABC algorithm is used, for each rate-of-growth scenario in turn, to construct an approximate posterior distribution of all the abundance and demographic rate parameters (see Methods section). The inputs are the posterior distributions from the acoustic-tag and CKMR models, plus prior distributions on fecundity and young-of-the-year survival. The key is to ensure that the implied rate-of-growth (given the combination of all those parameters in the Leslie matrix) is consistent with the specific rate-of-growth scenario being considered. Using the range of population decline/growth scenarios (*λ* = −0.04, −0.02, 0, 0.02 and 0.04), the median (and 95% credible interval) estimates for the total population size were 2,467 (1,464–4,381)3,161 (1,842–5,272), 4,064 (2,451–7,020), 5,375 (3,193–9,106), and 6,748 (4,138–11,083), respectively (Fig. 2(b)). The full set of results, before and after ABC, can be found in Table 1. Blending the models via ABC generally led to tighter confidence intervals on parameters, without changing the medians much.

While the close-kin data alone were not able to definitively estimate population rate of change, the results in Table 1 provide some insights into more probable levels of *λ* when accounting for juvenile and adult dynamics (both in terms of reproduction and survival). Scenarios of negative up to zero population growth (*λ* = −0.04, −0.02, 0) resulted in posterior estimates of juvenile survival that were most similar to their priors (themselves accurately estimated from the acoustic detection data). Scenarios with moderately positive population growth (*λ* = 0.02, 0.04) resulted in posterior estimates of juvenile survival that were the least similar to their priors, and resulted in updating of the priors for realised fecundity (*α*) and young-of-the-year survival multiplier (*δ*) parameters (see methods).

## Discussion

From our novel genetic approach, we estimate the size of the east Australian and New Zealand *adult* white shark population (males and females) to be 280–650 individuals. This approach also provided estimates of adult survival and indications of adult sex ratio. We combined these with estimated juvenile survival rates from acoustic telemetry and available life-history information (maturity and fecundity) to estimate total abundance in the region. Estimated total population size is 2,500–6,750, with survival rates for juveniles of around 70–75% and above 90% for mature adults. They provide the population size spanning the birth year range of the juvenile sharks sampled (1984–2013). These results are dependent on the known life-history constraints of the species and conditional on population growth scenarios bounded within the available data, but are concordant with the ecology of a long-lived, apex predator.

Robust estimates of the abundance, status and future outlook for large marine predators are vital for their conservation, understanding ecosystem function and understanding patterns of human-shark interactions world-wide. The current lack of such estimates for the most high-profile and now well studied and protected shark, the white shark, is testament to the difficulty in obtaining them with traditional approaches^16^. Our current data are not yet sufficient to estimate population trend, but ongoing sampling will resolve this as further acoustic and half-sibling data accumulate. The most we can say at present is that the current estimates of abundance and key demographic parameters (survival, fecundity etc.) are more consistent with slight current declines or zero population rate of change. The conservation implications of an adult population size in the several hundreds are unclear because the risk of small population sizes to long-term population viability for large marine predators, and for this white shark population in particular, is unknown. However, the high survival rates of juveniles and particularly adults imply that the population is comprised of many cohorts and, thus, expected to be relatively resistant to short-lived, random increases in mortality or decreases in reproductive success. Estimates of trend are needed before the impact of additional mortalities arising from fisheries bycatch and/or targeted captures via shark control programs aimed at reducing the risk of shark attack can be determined.

Other genetic-based methods for estimating adult abundance focus on generational effective population size, *N*_*e*_^31^, or the number of breeders *N*_*b*_^32^. Neither quantity relates directly to adult abundance (“census N” or *N*^*c*^) except in special cases, and both can be substantially smaller than *N*_*c*_ if, for example, there is strong variation in survivorship from litter to litter, or if offspring are not produced annually – often the case for elasmobranchs. Although some studies conclude that *N*_*e*_ estimates can approximate *N*_*c*_ in sharks in some circumstances^33^, overall the relationship between *N*_*e*_ and *N*_*c*_ can be period-dependent, influenced by the genetic history of the population, and generally unclear^34^. In particular, many estimators of *N*_*e*_ derived for overlapping generations^35–37^ have assumed no systematic trend in adult abundance, which may be inappropriate precisely for some species of most conservation concern. In contrast, *N*_*c*_ and trend are built in directly to the CKMR approach^20^ (see also Supp. Mat. S2.8.1). Of course, there are connections between *N*_*e*_ and CKMR, especially the sibling-based formulation used here. However, the CKMR equations need to be set up to account explicitly for any factors leading to systematic variation between adults in reproductive output, e.g. body size and variation in individual lifespan (accommodated through a mortality rate). Those same factors contribute implicitly to individual variance in whole-of-life reproductive output, which drives the *N*_*e*_-to-*N*_*c*_ ratio^37^. Another factor would be if a substantial proportion of adults are perpetually infertile; they would be invisible to a sibling-based CKMR study like ours, but we have assumed this is not the case for white sharks. Aside from trend and the explicit *vs*. implicit treatment of individual differences, perhaps the biggest difference between CKMR and *N*_*e*_ concerns random year-to-year variability in annual reproductive output (e.g. litter size), which can have a big effect on *N*_*e*_ but not on *N*_*c*_. The ensuing within-cohort siblings constitute the entire basis of Wang’s well-known method^32^ for estimating *N*_*b*_ in non-overlapping generations; for sibling-based CKMR, on the other hand, within-cohort sibship is a nuisance to be worked around (Supplement S2.5) and the focus is entirely on cross-cohort sibship, in such a way that random year-to-year variability becomes irrelevant. Effective population size (*N*_*e*_) and number of breeders (*N*_*b*_) do play an important role in assessing the genetic health of a population but, in our experience, from a conservation and management policy perspective it is usually *N*_*c*_ and the trend thereof which are the key quantities of interest.

The close-kin mark-recapture technique based on half-siblings enables the estimation of adult abundance when only juveniles can be easily sampled, a feature of several shark species and white sharks in particular^17^. A key assumption, which appears reasonable given white shark biology^2,7^, is that male and female reproductive output does not vary appreciably with size or age, though CKMR can potentially deal with that provided both juvenile and adult samples are available, using parent-offspring and half-sibling pairs^20^. Our method requires almost no external assumptions about demographic parameters, and is “self-correcting” (i.e. will improve as more data becomes available). Although the results so far are not very precise, that is merely a consequence of small sample sizes and a limited range of sampled cohorts. As data accumulates in future, we will be able to explore more refined models, and to improve the precision of estimates - in particular, of abundance trend.

Crucially, whatever the particulars of the sampling scheme in terms of the animal’s life-history, CKMR only requires tissue/DNA samples, which can often be secured non-lethally. This study also demonstrates the utility of large-scale coastal acoustic receiver arrays across national and international scales for the tracking of tagged marine predators^38^. Combining CKMR and acoustic telemetry over the geographic range of the population provides estimates of key demographic parameters needed to estimate total population size. The component methods outlined in this work, as well as their integration, are likely applicable to other white shark populations and similar species especially in areas with active electronic or conventional tagging programs and where simultaneous tissue sampling is logistically feasible. We recommend that all such programs take tissue biopsies as a matter of standard practice. Given the history of tragic interactions with humans, large marine predators like white sharks uniquely capture public attention. Ensuing debates regarding conservation, government and international policy implementation, and shark attack risk mitigation have lacked robust estimates of shark population size and trend, or even viable approaches for obtaining these. These debates will continue as human use of the oceans increases, and especially if white shark numbers also increase, the latter being the objective of conservation actions. The results presented here describe a realistic and achievable route for underpinning evidence-based conservation and risk-management decisions for this, and similar, species across the globe.

## Methods

### Genetic data

Section S1.1 in the supplementary material details the specifics of the data collection protocols and the processing of the nuclear and mitochondrial genetic data. In relation to the tagging of sharks, all methods were performed in accordance with the relevant guidelines and regulations relating to the jurisdictions in which the fieldwork was undertaken. Supplement S2.1–S2.3 explain the steps followed to identify half-sibling pairs along with quantified false-positive and false-negative rates, including allele frequency estimation, quality control, and kin-finding *per se*.

### Half-sibling demography model

Our analyses work in both an age and sex-independent modelling framework for the close-kin half-sibling data. Because we still have rather few HSPs, we have kept the population dynamics model simple; in particular, we have merged the two sexes, which could in principle be treated separately. We have also assumed that adult abundance trends were consistent (i.e. up, *status quo*, or down) during the period, and that any density-dependent changes in reproductive output were small. Most of our samples would have been born between around the year 1995 and 2010 (i.e. after protection was implemented).

Although our final model is sex-aggregated, it is simpler to start by explaining the ideas for just one sex of parent, e.g. for maternal half-siblings only. Following from the Introduction, assume for now that adult abundance is stable over time and that all females have equal annual reproductive output on average (though still random within any year). The probability that two juveniles *i* and *j* born a few years apart (say *δ* years) share the same mother is

2 $${p}_{mHSP}(i,j)={({\varphi }^{A})}^{\delta }/{N}_{\venus }$$

where the numerator is the chance that *i*’s mother is still alive when *j* was born (governed by an annual survival rate *ϕ*^*A*^), and the denominator reflects the chance that she also then happens to be the one female who is *j*’s mother, out of all the possible mothers that year.

A very similar formula applies to paternal half-siblings under similar assumptions, with *N*_♀_ instead of *N*_♀_. For a 50/50 sex ratio in the population (which was consistent with both the white shark mitochondrial DNA data and the sex ratio in the juveniles sampled) *N*_♀_ = *N*_♀_ = *N*^*A*^*/2*, where *N*^*A*^ is the total number of adults. For both sexes, the number of other potential parents of that sex is *2/N*^*A*^, so the probability that the pair share either a mother or a father is

3 $${p}_{HSP}=(4/{N}^{A})* {({\varphi }^{A})}^{\delta }.$$

To account for a changing population abundance over time, we assumed a minimal exponential growth model: *N*_*t*_ = *N*_*init*_ *e*^*λt*^, where *N*_*init*_ is the abundance in the initial model year. If *c*_*i*_ and *c*_*j*_ are the respective cohorts of the pair, and *c*_*i*_ is not equal to *c*_*j*_ (see below), the fully time-dependent HSP probability is as follows:

4 $${p}_{HSP}=(4/{N}_{{c}_{max}}^{A})* {({\varphi }^{A})}^{| {c}_{i}-{c}_{j}| },$$

and *c*_*max*_ = max{*c*_*i*_, *c*_*j*_} - i.e. it is the adult abundance *in the more recent cohort* that is relevant in the full time-dependent HSP probability. The three estimable parameters of this model are *N*_*init*_, *ϕ*^*A*^, and *λ*. A more detailed derivation, including the case when reproductive output changes through adulthood, is given in Supplement S2.8.1.

The log-likelihood of one pairwise comparison for half-sibship between animals *i* and *j* comes from a Bernoulli distribution:

5 $${\rm{lglk}}\,(i,j| {N}_{init},{\varphi }^{A},{\rm{\lambda }},{c}_{i},{c}_{j})={k}_{ij}\,{\rm{log}}\,{p}_{H}(i,j)+(1-{k}_{ij})\,{\rm{log}}\,(1-{p}_{HSP}(i,j))$$

where *k*_*ij*_ = 1 if the pair is an HSP or 0 if it is not. Multiple pairwise kinship comparisons are approximately statistically independent provided that the population is large enough compared to the number of samples^20^, so that the log-likelihoods from all comparisons can be summed to give an approximate overall log-likelihood from the CKMR model.

An additional source of uncertainty that must be accounted for is the cohort for each juvenile. The cohort is defined as *c* = *y*-*a*, where *y* is the year of sampling and *a* is the animal’s age. The sampling year is known without error here, but we do not have a direct measurement of age for most animals (e.g. from vertebral slices). For animals without direct age, we calculated their conditional probability distributions of age-at-capture given their lengths-at-capture, based on a separate dataset of vertebral age and length measurements for white sharks; see Supplement S2.4 for details. The uncertainty in cohort-ages for each pair can then be handled by marginalizing (6) across the conditional age distributions *p*^age^(*a*|*l*). Omitting the parameters for brevity, this gives:

6 $$\begin{array}{rcl}{\rm{lglk}}(i,j| {y}_{i},{y}_{j},{l}_{i},{l}_{j}) & = & \sum _{{a}_{i}}\sum _{{a}_{j}}{p}^{{\rm{age}}}({a}_{i}| {l}_{i}){p}^{{\rm{age}}}({a}_{j}| {l}_{j})\\  &  & \times exp[{\rm{lglk}}(i,j| {c}_{i}={y}_{i}-{a}_{i},{c}_{j}={y}_{j}-{a}_{j})]\end{array}$$

Section S2.5 describes how to deal with the complications that arise when the two animals may in fact from the same cohort, especially through random variability which can lead to more same-cohort siblings than equation (4) would suggest, and thus to bias if ignored. In our case, only about 5% of the pairwise comparisons are likely to be same-cohort, and we were able to circumvent any major bias with a simple post hoc adjustment. Section 2.8.1 considers whether our CKMR results might be sensitive to the assumption of knife-edge maturity in the CKMR model, as opposed to the maturity ogive used elsewhere in the paper; the potential impact turns out to be minimal, but the derivation of the equations provides insight into the phenomena that can affect sibling-based CKMR in general.

### Acoustic detection model

The model to estimate juvenile survival is a simple version of the Cormack-Jolly-Seber model^21^, where both juvenile survival, *ϕ*^*J*^, and the observation probability, *p*_*obs*_, are assumed time and age independent. Section S1.2 in the supplementary material has more detail on the acoustic data used in the juvenile tag survival model.

### Total population size model

The ABC algorithm uses the posterior distributions of adult survival from the close-kin (conditioned on a specific growth-rate scenario), and of juvenile survival from the acoustic mark-recapture model. The two remaining Leslie matrix parameters–effective fecundity (per year per female) α, and the age-0 survival multiplier *δ–*were assigned informative priors (see Section S2.6 of the supplementary material). Fecundity-at-age, *f*_*a*_, in the Leslie matrix, **Λ**, is defined via the product of effective fecundity (*α)* and maturity-at-age (*m*_*a*_), i.e. *f*_*a*_ = *αm*_*a*_. We also use the maturity curve, *m*_*a*_, to define the transition between the two survival estimates, for ages *a* ≥ 1:

7 $${\varphi }_{a}=(1-{m}_{a}){\varphi }^{J}+{m}_{a}{\varphi }^{A}.$$

We define an additional estimated parameter, *δ*∈[0, 1], and *ϕ*_0_ = *δϕ*_1_, allowing for lower, more variable, survival in young-of-the-year sharks for which there were no acoustic data. With the effective female fecundity parameter, *α*, this completes the demography required for the construction of the Leslie matrix, **Λ**.

The ABC algorithm samples from the joint prior distribution of the four parameters, ${\mathbb{P}}$(*ϕ*^*J*^*, ϕ*^*A*^*,α,δ*), and accepts this sample *only* if it results in a population growth rate (uniquely defined by the Leslie matrix configuration) within specified tolerance bounds. By repeating this process many times to obtain 1,000 samples we have effectively obtained a representative sample from the approximate posterior distribution of the parameters of interest. Readers are directed to the Supplement for further detail: Section S2.7 on development of the Leslie matrix; Section S2.7.5 for the specifics of the ABC algorithm itself; and Section S2.8 for sensitivity analyses relating to key structural uncertainties (no age effects, conflating of population and adult rate of change) likely to affect the overall population estimates.

The adult fraction, *χ*, of the total population, *N*, is

8 $$\chi =\frac{{\sum }_{a}{m}_{a}{\zeta }_{a}}{{\sum }_{a}{\rm{\zeta }}},$$

where *ζ*_*a*_ is the stable age distribution, defined by the lead eigenvector of **Λ**. Each sample of adult survival, *ϕ*^*A*^, has an associated value of adult abundance, *N*^*A*^ (from the CKMR analyses) and of the adult fraction, *χ*. The total population abundance for this sample is calculated as follows:

9 $$N={N}^{A}/\chi $$

## Electronic supplementary material

Supplementary material
