# Supplementary material for: Genetic relatedness reveals total population size of white sharks in eastern Australia and New Zealand
Source: Sci Rep. 2018 Feb 8;8:2661. doi: 10.1038/s41598-018-20593-w (PMC5805677; doi:10.1038/s41598-018-20593-w)
Supplement: Supplementary file 1 — Supplementary material [file 41598_2018_20593_MOESM1_ESM.pdf]

Genetic relatedness reveals total population size of white sharks in eastern Australia and New Zealand: supplementary material

R.M. Hillary, CSIRO M.V. Bravington, CSIRO T.A. Patterson, CSIRO
P. Grewe, CSIRO R. Bradford, CSIRO P. Feutry, CSIRO R. Gunasakera, CSIRO
V. Peddemors, NSW DPI J. Werry, Griffith University M. P. Francis, NIWA
C.A.J. Duffy, Department of Conservation, New Zealand B.D. Bruce, CSIRO

October 6, 2017

S1: Material and Methods

S1.1: Material

A total of 183 white shark tissue samples were genotyped for the purposes of this study. Samples were taken from both live and deceased animals. Live samples were taken in the process of placing satellite and acoustic tags on the sharks. The spatial extent of the samples was purposely designed to cover the observed range of the animal: all around the low tropical, sub-tropical, and temperate waters of both Australia and New Zealand (see Fig. 1 in main text for geographical spread). The earliest sample taken is from 1990, with the latest taken in 2015, though the vast majority come from the years 2000 to 2015. In terms of the birth-cohorts and, by implication, the years over which adult abundance is modelled, the minimum is 1984 and the maximum 2013, with most cohorts being from 1997 onwards. While full legal protection for white sharks in Australian waters did not start until the late 1990s, in practice the anthropogenic mortality rate off eastern Australia had likely been reduced substantially well before that, due to changes in recreational fishing effort and to spatial closures under commercial fisheries management in the early 1980s. . These included a move to offshore game-fishing in New South Wales where captures of white sharks were substantially less than inshore, and the introduction of ban on commercial gill netting in Victorian State waters that likely reduced the bycatch of juvenile white sharks in the SE Victorian nursery area. Given these factors we feel it is appropriate to include all the data in the model analyses.

S1.1.1: Sample genotyping

For this study DArTseq™ genotyping was used to process the samples. DArTseq combines DArT genomic complexity reduction methods with next-generation sequencing[43,44,45,4647,48]. Similar to DArT methods based on array hybridisations, the technology is optimized for each organism and application by selecting the most appropriate complexity reduction method (both the size of the representation and the fraction of a genome selected for assays). Four methods of complexity reduction were tested in tuna (data not presented) and the *Pst*I-*Sph*I method selected. DNA samples were processed in digestion/ligation reactions principally, but replacing a single *Pst*I-compatible adaptor with two different adaptors corresponding to two different Restriction Enzyme (RE) overhangs. The *Pst*I-compatible adapter was designed to include Illumina flow cell

attachment sequence, sequencing primer sequence and "staggered", varying length barcode region, similar to the reported sequence. The reverse adapter contained a flow cell attachment region and an *Sph*I-compatible overhang sequence.

Only "mixed fragments" (*Pst*I-*Sph*I) were effectively amplified by PCR. PCR conditions consisted of an initial denaturation at 94°C for 1 min followed by 30 cycles of 94°C for 20 sec, 58°C for 30 sec and 72°C for 45 sec, with a final extension step at 72°C for 7 min. After PCR equimolar amounts of amplification products from each sample of the 96-well microtiter plate were bulked and applied to cBot (Illumina) bridge PCR followed by sequencing on an Illumina Hiseq2000. The sequencing (single read) was run for 77 cycles.

Sequences generated from each lane were processed using proprietary DArTseq analytical pipelines. In the primary pipeline, the FASTQ files were first processed to filter away poor quality sequences, applying more stringent selection criteria to the barcode region compared to the rest of the sequence. In that way the assignments of the sequences to specific samples carried in the "barcode split" step were very reliable. Approximately 2,000,000 sequences per barcode/sample were identified and used in marker calling. Finally, identical sequences were collapsed into "fastqcoll files". The fastqcoll files were "groomed" using DArT PLD's proprietary algorithm that corrects low quality base from singleton tag into a correct base using collapsed tags with multiple members as a template. The "groomed" fastqcoll files were used in the secondary pipeline for DArT PLD's proprietary SNP and SilicoDArT (presence/absence of restriction fragments in representation) calling algorithms (DArTsoft14). For SNP calling all tags from all libraries included in the DArTsoft14 analysis are clustered using DArT PLD's C++ algorithm at the threshold distance of 3, followed by parsing of the clusters into separate SNP loci using a range of technical parameters, especially the balance of read counts for the allelic pairs. Additional selection criteria were added to the algorithm based on analysis of approximately 1,000 controlled cross populations. Testing for Mendelian distribution of alleles in these populations facilitated selection of technical parameters discriminating well true allelic variants from paralogous sequences. In addition, multiple samples were processed from DNA to allelic calls as technical replicates and scoring consistency was used as the main selection criteria for high quality/low error rate markers. Calling quality was assured by high average read depth per locus.

S1.1.2: Mitogenome sequencing

Total DNA used as template for mtDNA PCR amplification and DArT library construction was extracted from the tissue samples using a CTAB extraction protocol[49]. Whole mitogenomes were amplified in two overlapping fragments using Takara LA Taq, a proofreading Taq polymerase mixture (Takara, Otsu, Shiga, Japan). The PCR reaction mix consisted of 1 unit of Takara LA Taq, 2 μl of 10x LA PCR Buffer II (Mg2+ free), 2 μl of 25 mM MgCl2, 3.2 μl dNTP (2.5 mM each), 0.5 μl of each primer at 10 mM, and 10.6 μl sterilized distilled water.Amplification of the whole mitogenome was accomplished using two sets of primers to generate overlapping fragments, A and B (Table S1). The primer pairs were designed using previously acquired conserved areas of whole mitogenomic sequences of White shark[50], and the riversharks *Glyphis glyphis*[51] and *Glyphis garricki*[52]. PCR conditions consisted of an initial denaturation at 94°C for 3 min followed by 35 cycles of 94°C for 35 sec and 68°C for 10 min, with a final extension step of 72°C for 10 min. PCR amplicons for each individual were purified with Agencourt AMPure XP magnetic beads (Beckman Coulter Inc., Indianapolis, Indiana, USA), quantified with NanoDrop 8000 Spectrophotometer (Thermo Fisher Scientific, Waltham, Massachusetts, USA) and pooled at equimolar concentrations for each individual previous to the preparation of libraries.

The two purified fragments obtained for each individual were pooled at equimolar concentrations and

subjected to library preparation using Nextera XT DNA Sample Preparation kits (Illumina, San Diego, California, USA). Individual Nextera XT libraries were simultaneously fragmented and barcoded using the 96 sample Nextera Index kit (Illumina, San Diego, California, USA). All libraries were quantified by a fluorometric method, using the Qubit dsDNA HS assay kit (Life Technologies, Carlsbad, California, USA), and concentrations were normalized to give equal mitogenome coverage from each individual fish. Libraries were then pooled and sequenced on a Miseq desktop sequencer using 2x250 bp paired-end reads MiSeq reagent kit v2 (Illumina, San Diego, California, USA). The sequencing was done in two different runs of 96 individuals each.

Fastq files were imported into GENEIOUS PRO (v7.0.6) (Biomatters Ltd., Auckland, New Zealand). Before mitogenome assembly, reads were paired; 5' and 3' ends as well as regions with more than 5% chance of an error per base were trimmed. Reads with more than 15 low quality bases and/or shorter than 150 bp after trimming were discarded from subsequent analyses. Reads for each individual were then mapped onto a previously published reference sequence[52] using "Map to Reference" tool in GENEIOUS PRO without fine-tuning and default parameters.

PCR duplicates were removed using the rmdup SAMTools toolkit (v1.0.0)[53]. The majority rule consensus ($\geq$50% of reads for any single SNP, insertion, deletion) for each fish was exported. Consensus sequences were then aligned using the MUSCLE alignment tool with default parameters in GENEIOUS PRO. Two sets of primers (see Table S1) were used to amplify two overlapping fragments, A (8231bp) and B(9263bp). Standard amplification cycling conditions were used[53].

S1.2: Acoustic tag data collection and aggregation

Satellite tracking shows that juvenile white sharks range widely, but mostly in coastal waters, off Eastern Australia[24]. This data also (see Fig. 1 in the main text) indicates that almost all juveniles in the population congregate in the NSW nursery area from October-December before again dispersing. Consequently it is expected that the coastal distribution of juveniles and the wide range of the acoustic array, with particular high coverage within the juvenile nursery grounds off NSW, will yield robust data for estimation of juvenile survival. Over the years 2008 to 2014, juvenile white sharks were tagged in this area with Vemco (Vemco Ltd, Nova Scotia, Canada) acoustic tags. The locations of tagging are shown in Figure S1. Table S2 details the tag type, sex, location and number of the released tags; Table S3 details additional information relating to numbers, year and sex of release. The tags last reliably for many years, emit acoustic pulses at intervals of every few minutes which can be detected on fixed acoustic receivers such as the wide-scale acoustic array along the eastern Australian coast, maintained under the Australian Animal Tracking Facility which is part of Australia's Integrated Marine Observing System (IMOS, www.imos.org). This comprises over 300 Vemco VR2 receivers ranging from southern waters in Tasmania to North Queensland and around the Victorian and South Australian coasts. Similar acoustic tracking data from receivers in New Zealand waters were also included to increase coverage of the populations distribution. To match the population dynamics model acoustic detections were aggregated on an annual basis cross all receivers within each year to form individual annual capture histories (Table S4). Data were input into the Cormack-Jolly Seber model referred to in the main text which had fixed parameters of detection probability and annual survival. This model was estimated using a Bayesian framework[54] and fitted using the Bayesian estimation software `Stan`[55]. The estimates in Table S5 indicated a moderately high value of annual survival (ϕ^J) which is plausible for a top-predator which attains a large size even as a juvenile. The high estimate of detection probability indicating that juveniles are highly likely to be detected somewhere within their range and therefore, that the large

scale array is a highly useful tool for gathering information on this important demographic parameter.

S2: Methods

S2.1: Estimating allele frequencies

Genuine null alleles (i.e. occuring consistently across repeated genotyping, as opposed to being artefacts of low read-depths) are quite common in our white shark DArTseq™ data; they arise presumably from polymorphism at either of the two restriction sites for each locus, and/or from indels that change the length of some alleles. Detailed examination of our kin-pairs shows that the nulls are heritable just like regular SNPs.The raw genetic data therefore correspond to four possible observable genotypes for each fish at a given locus: AA0, AB, BB0, and 00. Here A and B are the usual alleles, 0 denotes a null allele, and AA0 means "either AA or A0" since those possibilities cannot be distinguished. Two parameters per locus are estimated within the model: (i) π_A, which is the conditionally non-null probability of allele A; and (ii) π_0 which is the probability of a null allele. The probabilities of the four observed genotypes are:

$$\begin{aligned}
p_{AA0} &= \left(\pi_A \left(1-\pi_0\right)\right)^2 + 2\pi_A \left(1-\pi_0\right)\pi_0, \\
p_{AB} &= 2\pi_A \left(1-\pi_A\right)\left(1-\pi_0\right)^2, \\
p_{BB0} &= \left(\left(1-\pi_A\right)\left(1-\pi_0\right)\right)^2 + 2\left(1-\pi_A\right)\left(1-\pi_0\right)\pi_0, \\
p_{00} &= \pi_0^2.
\end{aligned}$$

For numerical stability, the actual estimated parameters are the logit-transformed counterparts of π_A and π_0 and the likelihood function (for each locus) is the product of categorical multinomial distributions:

$$\mathbb{P}\left(\mathbf{G} \mid \cdots\right) = c\left(\mathbf{G}\right) p_{AA0}^{n_{AA0}} p_{AB}^{n_{AB}} p_{BB0}^{n_{BB0}} p_{00}^{n_{00}}$$

where $\cdots$ denotes the two allele frequency parameters, $\mathbf{G}$ is the set of genotypes of all the fish at that locus, $c\left(\mathbf{G}\right)$ is a constant which does not depend on the allele frequency parameters, and n_g denotes the number of fish of genotype g at that locus. Initially, a high proportion of loci are estimated to have very low minor allele frequencies (MAF) and are going to be uninformative. While the subsequent calculations of relatedness are robust to retaining such loci, it is hard to check reliability of loci with extreme frequencies, so an initial subset of loci were retained ($\pi_A \in [0.2, 0.8]$ and $\pi_0 \in [0, 0.3]$) which left 3,107 of the original 8,961 loci.

In conservation studies where limited numbers of animals are sampled available, as in this paper (115 in this case, after the QC steps below), uncertainty in the allele frequencies needs to be accounted for. Asymptotic methods are unreliable here because it is perfectly permissible for the parameter estimates to hit bounds (e.g. if there is no evidence of null alleles, so that $\pi_0 = 0$), at which point Hessian matrix-based covariance estimates are either unobtainable or unreliable. Given the very large number of loci (around 8,000) the bootstrap was employed:

1. Independence of the loci was assumed (i.e. negligible linkage disequilibrium— a reasonable assumption with just a few thousand loci across a 6.4Gb genome)

2. For each locus ℓ, the genotype set $\mathbf{G}_\ell$ was resampled via the bootstrap

3. For each resample $\mathbf{G}_\ell^{[b]}$ at each locus, $\hat{\pi}_{A\ell}^{[b]}$ and $\hat{\pi}_{0\ell}^{[b]}$ were estimated via maximum likelihood
4. This process was repeated to give 1,000 bootstrap pairs of estimates $\left\{\hat{\pi}_{A\ell}^{[b]}, \hat{\pi}_{0\ell}^{[b]};\ b \in 1 \cdots 1000\right\}$ for the locus

S2.2: Quality control and removal of duplicate samples

A number of quality control steps were taken before identification of sibling-pairs for use in the population model. Firstly, the adequacy of each locus was assessed in terms of how well the estimated allele frequencies predicted the observed heterozygosity across fish: i.e., whether the locus appeared consistent with the assumptions underlying Hardy-Weinberg Equilibrium, as needed later for inferring kinship. Then each individual sample was examined in terms of its observed heterozygosity across all loci. Next, any duplicate fish were removed, including both repeat samples and genuine secondary "recaptures" of fish.

S2.2.1: Predictive performance across loci

Treating the bootstrap samples of $\left\{\hat{\pi_A}_\ell^{[b]}, \hat{\pi_0}_\ell^{[b]}\right\}$ as draws from an approximate posterior distribution for locus ℓ, we use posterior predictive analysis to assess the performance of the locus[56]. Our kin-finding algorithms (S2.3) require that all loci satisfy the assumptions required for Hardy-Weinberg equilibrium, so we focus on comparing the predicted heterozygosity rate at each locus to the observed rate and across all fish. If $N_{AB;\ell}$ is a random variable denoting the potential number of heterozygous fish at a given locus ℓ, then we compute the following (Bayesian) p-value:

$$p_\ell^{\text{hetz}} = \mathbb{P}\left(N_{AB;\ell} > n_{AB;\ell}\right),$$

where $n_{AB;\ell}$ is the observed value. The distribution of $N_{AB;\ell}$ is Binomial with maximum value the number of fish, and probability $p_{AB;\ell} = 2\pi_{A\ell}\left(1 - \pi_{A\ell}\right)\left(1 - \pi_{0\ell}\right)^2$ conditional on the allele frequencies, but the latter are uncertain. We can therefore simulate a draw $N^*_{AB;\ell}$ from the overall distribution by first drawing the two allele frequencies from the bootstrap samples $\left\{\hat{\pi_A}_\ell^{[b]}, \hat{\pi_0}_\ell^{[b]}\right\}$, and then generating a Binomial deviate. Finally, p_ℓ^{hetz} can be computed from the proportion of such $N^*_{AB;\ell}$ that exceed $n_{AB;\ell}$.

For loci in Hardy-Weinberg equilibrium, the distribution of p^{hetz} across loci should be uniform over the unit interval. In this case, there were a number of loci that gave values very close to (and sometimes equal to) zero and one, forming noticeable modes at the boundaries. These loci were removed so that the remaining distribution of p^{hetz} looked suitably uniform, leaving 2,186 loci.

S2.2.2: Predictive performance across samples

A similar analysis is used to find anomalous samples, in the sense that their genotype does not correspond well with that predicted by the model. The main concerns here were poor DNA quality (which can lead to alleles being overlooked, and thus a deficiency of apparent heterozygotes) and tissue contamination (which can lead to excess heterozygotes). For a "well-behaved" sample, the heterozygosity status at locus ℓ should be a Bernoulli random variable (i.e. a Binomial random variable with maximum value 1) with probability $p_{AB;\ell} = 2\pi_{A\ell}\left(1 - \pi_{A\ell}\right)\left(1 - \pi_{0\ell}\right)^2$. To get the probability distribution of the number of heterozygous loci for a fish, we need to sum across all these individual Bernoulli distributions. Because each has its own its distinct probability, the sum is no longer binomial as it was for locus-checking, but we can still draw from its distribution using the Butler-Stephens algorithm[57], which defines the exact distribution of the sum of

differing-probability binomial (including Bernoulli) random variables[53]. We then define our heterozygosity p-value for sample i as

$$p_i^{\text{hetz}} = \mathbb{P}\left(N_{AB;i} > n_{AB;i}\right),$$

where $N_{AB;i}$ is the potential number of heterozygous loci and $n_{AB;s}$ is the observed number. As in the locus-checking algorithm, both the estimation uncertainty in the allele frequency model parameters and the sampling variability in the genotypes at each locus was accounted for. Samples with values on the extrema of the unit interval were removed to obtain a uniform-like distribution, leaving 115 of the 183 original samples.

S2.2.3: Removal of duplicates

Repeat samples were simple to find via pairwise comparisons, based on the proportion of loci having identical genotypes in the pair(with 115 fish, that made 6,555 comparisons). A total of 9 pairs had close to 100% (98-99% in general) identical genotypes, and stood out clearly from the vast majority of non-duplicate pairs. There were no multiple duplicates- no sample in one duplicate pairappeared in any another - so one sample from each duplicate pair was randomly chosen for removal, to leave a dataset of 106 distinct fish.

S2.3: Identification of sib-pairs

Our CKMR model is built around half-sib pairs in different cohorts. Half-sibship leaves a rather weak genetic signal whose strength varies from pair to pair. Special care is needed to control false-positives from unrelated and more-weakly-related pairs, which in turn means that the proportion of false-negatives needs to be estimated; as explained in S2.3.5, the latter requires a set of unambiguous HSPs. The potential for full-sib pairs to comp licate the search for true half-sib pairs is clear, so we tackle the process in two stages. First we find pairs of full-sibs, a strong relationship which is unambiguously visible given appropriate statistical processing. After removing one full-sib from each FSP, we then look for HSPs among the remaining animals, including allowance for false-positives and false-negatives. The statistical approach is similar in both stages, using a likelihood-ratio framework which follows standard principles of statistical genetics, adapted to the nature of our DArTseq™ data. For clarity, we describe the full-sib version first; the modifications for half-sibs are described in S2.3.3.

For each pair being compared, at each locus, we compute the probability of the two observed genotypes under two possible kinships for the pair: full-sib or unrelated. We then take the ratio of the two probabilities (a likelihood ratio), log it, repeat for all loci, and then average the log-ratios to get an overall statistic (the “PLOD”) for that pair. Broadly speaking, a large positive value is evidence in favour of full-sibship, and a negative value is evidence against. We next give more detail on the likelihood ratios.

S2.3.1 Likelihood ratios for kinship

Consider a comparison between one pair of animals at one locus. The genotype of each animal at that locus could take any of the 4 observable values (AA0, AB, BB0, 00), and we refer to the two genotypes together as a “genopair”.

The genopair will contain 0, 1, or 2 identical-by-descent (IBD) alleles, i.e. inherited from a shared ancestor. Conditional on that number, the probabilities of the 16 observable genopairs can be expressed as 4x4 matrices M_0, M_1, and M_2, respectively, whose computation is described shortly. The actual kinship of the pair does not affect $M_{0/1/2}$, but it does affect the probability distribution of the number of IBD alleles,

which is $k_{\tilde{r}} = \{k_{\tilde{r}0}, k_{\tilde{r}1}, k_{\tilde{r}2}\}$ where $k_{\tilde{r1}}$ is the probability of having 1 IBD allele for kinship $\tilde{r}$, etc. For an unrelated pair, $k_{\text{up}} = \{1, 0, 0\}$; for full-siblings, $k_{\text{fsp}} = \{\frac{1}{4}, \frac{1}{2}, \frac{1}{4}\}$; and for half-siblings, $k_{\text{hsp}} = \{\frac{1}{2}, \frac{1}{2}, 0\}$. From basic probability theory, the overall matrix of genopair probabilities for a particular kinship $\tilde{r}$ is then a linear combination:

$$M^{\tilde{r}} = k_{\tilde{r}0}M_0 + k_{\tilde{r}1}M_1 + k_{\tilde{r}2}M_2,$$

.

We next explain how the matrices M_0, M_1, and M_2 are calculated from the allele frequencies at the given locus; note that we have ignored genotyping errors, since these are demonstrably rare in our dataset (<2% based on repeat genotyping) and have not distorted the good match we found between theory and empirical results (S2.3.4). A few definitions will make this task easier:

$$\begin{aligned}
p &= \pi_A(1 - \pi_0), \\
q &= (1 - \pi_A)(1 - \pi_0), \\
r &= \pi_0, \\
\mathbf{p} &= \{p^2 + 2pr, 2pq, q^2 + 2qr, r^2\}.
\end{aligned}$$

and $\mathbf{p}$ contains the probabilities of the four possible genotypes of a single animal, in the order AA0, AB, BB0, 00. For the matrix M_0, where zero alleles are IBD, then $M_0 = \mathbf{p} \otimes \mathbf{p}$ (the outer product of $\mathbf{p}$) because the two genotypes are independent. For M_2, where both alleles are IBD, the second genotype must be the same as the first, so the matrix has $\mathbf{p}$ on the diagonal and zero elsewhere. The only complicated case is M_1, where one allele is IBD but the second allele in each of the two animals is an independent random variable. Some algebra leads to this:

$$M_1 = \begin{pmatrix} p^3 + 3p^2r + r^2p & p^2q + pqr & pqr & pr^2 \\ p^2q + pqr & pq^2 + p^2q & pq^2 + prq & 0 \\ pqr & pq^2 + prq & q^3 + 3q^2r + qr^2 & qr^2 \\ pr^2 & 0 & qr^2 & r^3 \end{pmatrix}$$

We now consider how to use the observed genopairs at all loci to infer the likely kinship of the pair. This is based on the log-likelihood ratio, or "LOD score" (used in a general statistical sense, not related to genetic linkage studies), at any given locus: i.e., the log of the ratio of the probabilities of the observed genopair under the two working hypotheses about the kinship, namely full-sib or unrelated. To combine across loci, we take the sum (actually, the mean) of the locus-specific LODs. If the genopairs at different loci were statistically independent given the kinship, then the sum would be a true overall log-likelihood-ratio or LOD score for testing the kinship hypothesis. However, because of the large number of loci needed to find half-sibs, each chromosome will contain many loci and so, within related pairs of animals, there will be substantial correlations between the number of IBD alleles among nearby loci— i.e., there will be substantial linkage[21]. This does not invalidate the use of the summed LOD scores as a statistic for finding types of kin, but does mean that the per-locus LODs are no longer independent and that we are dealing instead with a pseudo-log-likelihood or Pseudo-LOD score. We therefore use the term "PLOD" for the mean LOD scores of each pair.

For a pairwise comparison of fish i and j at L loci in all, , where $g_\ell(i)$ is the genotype of i at locus ℓ, the PLOD is computed like so:

$$\mathrm{PLOD}_{\mathrm{up}}^{\mathrm{fsp}}\left(i,j\right)=\frac{1}{L}\sum_{\ell=1}^{L}\log\left(\frac{M^{\mathrm{fsp}}\left[\ell,g_{\ell}\left(i\right),g_{\ell}\left(j\right)\right]}{M^{\mathrm{up}}\left[\ell,g_{\ell}\left(i\right),g_{\ell}\left(j\right)\right]}\right)$$

The labels $\mathrm{PLOD}_{\mathrm{up}}^{\mathrm{fsp}}$ show which particular kinships are under consideration; later, a different version of PLOD is used for finding half-sibs. The ℓ-index on M indicates that each 4x4 matrix is specific to one locus.

Some distributional properties of the PLOD can be calculated easily, to serve as benchmarks both for validating assumptions and for making decisions about kinship based on individual PLODs. For pairs that are truly UP, the expected value of the PLOD is given by the following:

$$\mathbb{E}\left(\mathrm{PLOD}_{\mathrm{up}}^{\mathrm{fsp}}|\mathrm{UP}\right)=\frac{1}{L}\sum_{\ell=1}^{L}\sum_{g}\sum_{g'}M^{\mathrm{up}}\left[\ell,g,g'\right]\log\left(\frac{M^{\mathrm{fsp}}\left[\ell,g,g'\right]}{M^{\mathrm{up}}\left[\ell,g,g'\right]}\right)$$

where g and g'range over the set of observable genotypes {AA0,AB,BB0,00}. For pairs that are truly FSP, the expected value is

$$\mathbb{E}\left(\mathrm{PLOD}_{\mathrm{up}}^{\mathrm{fsp}}|\mathrm{FSP}\right)=\frac{1}{L}\sum_{\ell=1}^{L}\sum_{g}\sum_{g'}M^{\mathrm{fsp}}\left[\ell,g,g'\right]\log\left(\frac{M^{\mathrm{fsp}}\left[\ell,g,g'\right]}{M^{\mathrm{up}}\left[\ell,g,g'\right]}\right).$$

For true UPs, the PLOD variance can also be computed in the same way, because the PLOD is a sum across loci and the loci are independent for UPs (note that we are not using enough loci for Linkage-Disequilibrium to be a significant concern[21]). However, this cannot be done for related pairs because of linkage.

In principle, calculations of PLODs and expected values should perhaps take into account the uncertainty in estimated allele frequencies. However, while the latter is important for the predictive performance analyses in S2.2, we found that it had negligible impact on pairwise kinship comparisons. For simplicity, we therefore treated allele frequency estimates as exact in our PLOD calculations.

For a UP the expected PLOD will be negative, and for a FSP it will be positive. This follows from the properties of the Kullback-Liebler divergence of two probability distributions $P(\mathcal{I})$ and $Q(\mathcal{I})$ defined on a discrete space $\mathcal{I}$:

$$D_{KL}(P||Q)=\sum_{i\in\mathcal{I}}P(i)\ln\left(\frac{P(i)}{Q(i)}\right)\geq 0.$$

The expected LOD for each locus is evidently a Kullback-Liebler divergence, where $\mathcal{I}$ is the set of 16 genopairs, $P(\mathcal{I})$ is the genopair probability distribution for FSPs, and $Q(\mathcal{I})$ is the genopair probability distribution for UPs at the locus. So, by the properties of the Kullback-Liebler divergence, we must have that $\mathbb{E}\left[\mathrm{PLOD}_{\mathrm{up}}^{\mathrm{fsp}}|\mathrm{FSP}\right]>0$ (strictly positive because $P\neq Q$) and thus $\mathbb{E}\left(\mathrm{PLOD}_{\mathrm{up}}^{\mathrm{fsp}}|\mathrm{FSP}\right)>0$ by summation.

Conversely, when calculating the expected PLOD for the UP case, we actually calculate a mean across loci of the following:

$$f(P,Q)=\sum_{i\in\mathcal{I}}P(i)\ln\left(\frac{Q(i)}{P(i)}\right).$$

Using the Gibbs inequality, which determines the non-negativity of the Kullback-Leibler divergence, in a slight different way we can say the following:

$$-\sum_{i}P(i)\ln P(i)\leq-\sum_{i}P(i)\ln Q(i),$$

and moving the term of the r.h.s. of the above equation reduces to the definition of $f(P, Q)$ and is, clearly, non-positive and strictly negative if $P \neq Q$. This is why $\mathbb{E}\left(\mathrm{PLOD}_{\mathrm{up}}^{\mathrm{fsp}} | \mathrm{UP}\right) < 0$.

S2.3.2: Full-sib results

By calculating $\mathrm{PLOD}_{\mathrm{up}}^{\mathrm{fsp}}(i, j)$ for all (i, j) pairs amongst the 106 fish, i.e. 5,565 unique pairwise comparisons, it was clear that: (a) the vast majority of comparisons are UPs as expected (they are located around the UP mean); (b) 4 FSPs stand out quite clearly and are located around their expected value; (c) quite a few comparisons appear to be somewhat related, located well away from the UP mean but still distinct from the FSPs. Point (c) is taken up in the next section as these fish are highly likely to be half-siblings or perhaps first cousins. Based on lengths and capture-dates, each FSP seems likely to be intra-cohort; this is not surprising, since full-sibs from different cohorts would require repeat matings between the same male and female in multiple years, which will be uncommon in a reasonably large populations. No fish appears in more than one FSP. To proceed with HSP-finding, one fish from each FSP is randomly selected and removed from the dataset, leaving 102 fish; see also section S2.5 for a discussion of the implications of this removal.

S2.3.3: Finding HSPs

The same approach is used to identify any HSPs in the dataset— which are, ultimately, what we need for population modelling. The PLOD statistic in this case is very similar to that used in the FSP identification:

$$\mathrm{PLOD}_{\mathrm{up}}^{\mathrm{hsp}}(i, j) = \frac{1}{L} \sum_{\ell=1}^{L} \log \left(\frac{M^{\mathrm{hsp}}\left[\ell, g_{\ell}(i), g_{\ell}(j)\right]}{M^{\mathrm{up}}\left[\ell, g_{\ell}(i), g_{\ell}(j)\right]} \right),$$

with the expected values for true UPs and HSPs, and the variance for UPs, computed as per S2.3.1. From this point onwards, we use the unscripted term PLOD to always imply $\mathrm{PLOD}_{\mathrm{up}}^{\mathrm{hsp}}$ as computed for an HSP-vs-UP comparison, regardless of the true relationship of the pair.

To arrive at a reliable selection of HSPs and minimize any bias in abundance estimation arising from kinship uncertainty, we aim to keep false-positives very low, and then to infer the extent of any bias from false-negatives, according to the following steps; see elsewhere[21] for further details.

1. The mean and variance of the PLOD for UPs can be predicted from allele frequencies. Validate these against the empirical distribution, to check for linkage disequilibrium or other problems.

2. Choose cutoff PLOD based on mean and variance from UPs, to ensure that no more than a small proportion of pairs with PLODs above the cutoff are actually false-positives from UPs.

3. Estimate the variance of the PLOD for HSPs empirically, using a subset of definite HSPs with PLODs well above the cutoff; this is necessary to allow for the effects of linkage (which does not affect UPs).

4. Use the inferred extent of linkage in HSPs to roughly predict the number of first-half-cousins (C1Ps, i.e. with one shared grandparent) that would fall above the cutoff. If necessary, increase the cutoff so that overall false-positive rate from all non-HSPs remains negligible.

5. Estimate the proportion of true HSPs likely to have PLOD below the cutoff (i.e. false negatives), using the estimated HSP PLOD variance. If necessary, apply a corresponding bias correction to overall abundance.

Figure S2 details the PLODs for each pairwise comparison, the expected values for true UPs and HSPs, and the relevant cutoffs required for false-positive exclusion. There are clearly a number of HSPs present (sitting above the expected value and well away from the UP zone) but, unlike with FSPs, there is no clear gap separating HSPs from UPs and less-related pairs.

Note also that it is not normally possible to genetically distinguish HSPs from grandparent-grandchild-pairs or full-thiatic-pairs (aunt-nephew etc)[21]; if such pairs were present, they would fall in exactly the same region of Figure S2 as true HSPs. However, all our sampled white sharks were immature, and many were dead; given the age-of-maturity of white sharks, lack of pair-bonding, and the ages and collection-years of our dataset, these other potentially-confounding strong kinships are not relevant to our study. Weaker kinships such as cousins are demographically certain to be present, and are dealt with in step 4.

S2.3.4: Cutoff selection and model validation (steps 1 and 2)

Using the importance sampling approach in[58] extended to include the uncertainty in the estimated allele frequencies, the PLOD value above which no more than 1 UP is likely (among 5,050 comparisons) is -0.012 (slightly above the expected PLOD for C1Ps). To validate our process against the observations, we compared the empirical mean PLOD for UPs μ_{emp} (calculated from PLODs less than -0.012) to the theoretical mean PLOD for UPs defined in section S2.3.2. The sampling variance of μ_{emp} is calculated using leave-one-out cross-validation of fish. Then the following statistic is calculated

$$\frac{\mu_{\text{emp}} - \mathbb{E}(\text{PLOD}_{\text{up}}^{\text{hsp}}|\text{UP})}{\sqrt{\mathbb{V}\left(\mu_{\text{emp}}\right)}},$$

to see if the theoretical mean is consistent with the observations. The value was 0.67 so the theoretical mean was within one sampling standard deviation of the empirical mean. The final check was to compare the empirical and theoretical variances in the PLOD for the UPs. The estimate of the empirical standard deviation was 0.0067, compared with 0.0063 for for the theoretical standard deviation.

Given that the theoretical and empirical means and variances are very close, and statistically indistinguishable, the underlying assumptions appear to be met; we feel confident that the model is predicting the distribution of the PLOD for the UPs well, and thus that the false positive calculations are likely to be robust and reliable.

S2.3.5: Estimating the variance of PLOD for true HSPs (step 3)

The slight complication with this step is that, although Figure S2 clearly reveals some HSPs, the dots above the UP cutoff cannot be assumed to be a random selection from true HSPs; some might actually be cousins, and also some true HSPs may have PLODs below the UP cutoff (i.e. false negatives). Fortunately, simulations suggest that the distribution of PLOD for HSPs should be quite symmetric even in the presence of linkage, and indeed fairly close to Normal— perhaps unsurprisingly in view of the large number of loci used. Also, any pair with a PLOD above the expected value for HSPs is almost certain to be a true HSP (since we have eliminated FSPs already, and all other demographically-plausible kinships are weaker). Then, if $\mathcal{H}^+$ is the set of such pairs and there are $n_{\mathcal{H}^+}$ of them, we can take advantage of the symmetry to form a simple unbiased estimate of $v^{\text{hsp}} = \mathbb{V}\left[\text{PLOD}|\text{HSP}\right]$ like so:

$$\hat{v}^{\text{hsp}} = n_{\mathcal{H}^+}^{-1} \sum_{i,j \in \mathcal{H}^+} \left(\text{PLOD}\left(i,j\right) - \mathbb{E}\left[\text{PLOD}|\text{HSP}\right]\right)^2$$

We thereby estimated a standard deviation of 0.011 for the PLOD of HSPs, compared to a theoretical value of 0.005 if there was no linkage. Assuming that PLODs for HSPs are approximately Normal, $\hat{v}^{\text{hsp}}$ can then be used to predict how many true HSPs might be lost as false-negatives below any particular cutoff.

S2.3.6: Excluding first cousins from the likely HSPs (step 4)

First (half-)cousins (C1Ps, i.e. with one shared grandparent) are a potential source of false-positives. They should be more common demographically than HSPs (though much rarer than UPs), and will tend to have higher PLODs than UPs (though much lower than HSPs). The expected PLOD for a C1P can be calculated from allele frequencies without any assumptions about linkage; here it is -0.015, only slightly below the PLOD cutoff of -0.012 chosen to exclude false-positive UPs. Inspection of Fig. S2 indeed reveals numerous pairs slightly above that cutoff.

The exact PLOD variance for C1Ps will lie between that for UPs and that for HSPs (v^{hsp}), depending on details of crossovers and chromosomal arrangement which are not known; it can be bounded (but not computed exactly) by a calculation that involves $\hat{v}^{\text{hsp}}$. Based on the upper-bound variance for C1Ps, assuming both a normal distribution for the PLOD statistic and a typical ratio of four C1Ps for each HSP, we adopted a cutoff of -0.002 (the bottom of the grey bar in Fig. S2) which should remove almost all false-positives from C1Ps; it is well above the cut-off for UPs alone.

S2.3.7: Remaining sources of bias

There are two remaining sources of bias, in opposing directions, which we have chosen not to address yet given the fairly small number of HSPs found to date (on which any bias correction has to be based), and the likely modest effects of these biases compared to the estimated CVs. However, both could be handled straightforwardly within the above framework given somewhat larger sample sizes and improved genetic precision (more loci etc.), and we expect to do so in future.

First, we have not compensated for expected false-negatives, i.e. true HSPs whose PLOD falls below the cutoff. False-negatives would give rise to an upward bias in abundance estimates. Based on the estimated PLOD variance for HSPs and the current cutoff, less than 5% of true HSPs have probably been lost to false-negatives, though this figure is quite sensitive to the estimated HSP PLOD variance which itself is based on a sample size of 9.

Second, we have not allowed for false-positives from Half-Thiatic-Pairs (HTPs: grandparent of one animal being the parent of the other) which, though demographically rarer than cousins, may also be reasonably common in a very long-lived species. HTPs are more related on average than C1Ps, though less so than HSPs. In the current data (for the Eastern population *only* and for animals with length-at-sampling data) there are slightly fewer nominal HSPs above the expected PLOD value for HSPs, versus below (but above the cutoff): 9 vs 11. While clearly not statistically significant, this difference would be consistent with a modest amount of contamination from HTPs, corresponding to some downwards bias in abundance estimates. In principle, such bias could be eliminated by raising the cutoff sufficiently but, given the limited number of potential HSPs, the bias-variance trade-off would currently be unfavourable.

S2.3.8: Final set of HSPs

The full dataset consisted of fish sampled from all around Australia and from New Zealand. Recent work has shown the high likelihood of two populations of white sharks in the region[22]. In our analyses, we found no full

or half-sibling pairs that appeared to cross the suggested "boundary" between the populations (essentially the middle of Bass Strait between the coast of the state of Victoria and the island state of Tasmania). Inferring a lack of mixing from traditional population genetics approaches and from close-kin type analyses are very different things. All we state here is that the close-kin information seems to support the theory that there is not a significant amount of mixing occurring in either the adult population — or the resulting juveniles — of the proposed Eastern and Western populations.

For the Western population, there were far fewer fish, and as a result not enough HSPs - two - to do any type of actual abundance estimate, so the primary focus was placed on the Eastern population. The 75 fish that came from the East of Australia and New Zealand were analysed in isolation to the Western fish for the purposes of deriving the data for the population model. Of a total of 2,775 comparisons between Eastern fish, using the procedures and cutoff PLOD level described in the previous section, there were 20 HSPs found in the "Eastern" population data set (with false positive/negative rates suggesting that this number is accurate to about $\pm$ 1 HSP)

S2.4: Dealing with age and length in the population model

In order to calculate the demographic probability that a pairwise comparison between two particular fish will yield an HSP, it is necessary to consider the difference between their respective birth-years (cohorts) . The cohort of a given fish is the year in which it was sampled, y, minus the age of the fish in that year: $c = y - a$. For our purposes, y is known without error but we do not have direct ages from each fish; we must infer the probability distribution of age given length (which was measured in each case) for each fish that was not aged directly. For the 75 fish in the final dataset, only 8 had direct age measurements.

S2.4.1: Estimating the distribution of length-at-age

There are a total of 69 historical[24] measurements of length-at-age for the white shark population we are analysing. For each of these fish their length was recorded and their age estimated from counting annual rings deposited in the verterbrae of the sharks (the most common way to age elasmobranchs). We are interested specifically in the length-at-age distribution of fish in the dataset, not of the whole length range of the population, so we take a non-parametric approach to estimating length-at-age for the range of lengths of interest (i.e. under 4m). The other good reason for not taking a more traditional approach is the absence of large/old fish in the dataset. This makes it very hard to estimate asymptotic length-at-age, which has a knock-on effect to other key parameters like initial length and growth rate. By excluding fish of length more than 4m we are left with 60 measurements of the length of a fish, given its estimated age.

A hierarchical Bayesian approach is used to fit a neural network (using radial basis functions, here denoted by φ) to the length-at-age data. The base model for mean length, l, in terms of age, a, is as follows:

$$\mathbb{E}[\ln l(a)] = \sum_{i=1}^{N} \omega_i \varphi \left(a, \mathbf{x}_i, \gamma\right),$$

where ω_i are the network weights, $\mathbf{x}_i$ are the network nodes, γ is a scaling parameter, and $N = 10$ is the number of network nodes. With the assumption of a log-normal distribution, the final term that defines the distribution of length-at-age (not just the expected length-at-age) is the process error variance, σ^2_{pe}, given a pre-specified observation error variance (i.e. variance in the log-lengths of sharks at a given age) of $\sigma^2_{\text{oe}} = 0.025$. Within the log-normal likelihood function for the length-at-age data, the overall variance

is defined as the sum of the observation and process variances. It is both the model for the mean length-at-age as well as the process error variance (and assumed log-normal likelihood function) that defines the distribution of length-at-age in the population, $p(l \mid a)$.

The main estimated parameters are the network weights, ω_i, which are assigned a normal prior with mean 0 and variance σ^2_ω. The hierarchical structure comes in because we also estimate the prior variance of the weights, σ^2_ω, which controls the flexibility of the neural network. The specifics of the functional form assumed for the network basis (multi-quadric), definition of the network nodes and weighting parameter γ, and the estimation scheme can be found in[59]. The Gibbs sampler is used to draw 101,000 samples from the joint posterior $\pi(\omega_i, \sigma^2_\omega \mid \cdots)$, the first 1,000 samples are discarded as a burn-in period and remaining 100,000 samples are thinned at a factor of 100 to obtain 1,000 samples from the relevant posterior. Standard convergence criteria[56] were applied to the resulting Markov chains and were adjudged to be usable in this regard. Figure S3 shows the posterior distribution of mean length-at-age as well as the posterior predictive performance of the model[56] using the median absolute residual deviation as the discrepancy statistic.

From Fig. S3 the mean length-at-age distribution clearly captures the relationship fairly well, and the posterior predictive p-value of 0.752, while suggesting the predictions are slightly more variable than the observations, does not show any major issues with how we are explaining the data either. Given these results, we use this model as the basis for defining the posterior distribution of length given age: $p(l \mid a)$.

S2.4.2: Estimating the prior age distribution in the dataset

Given the distribution of length-at-age, to obtain the distribution of age-at-length amongst our samples (which we what really need), we apply Bayes' rule:

$$p(a \mid l) = \frac{p(l \mid a)p(a)}{p(l)},$$

but to do this we need to define the prior, $p(a)$, on the age distribution of *sampled fish* (not of the population as a whole). We estimate the prior from the length composition of the sampled fish, using the following equation:

$$p(l) = \sum_a p(l \mid a)p(a).$$

Given the size of sharks in the dataset (from 1.3m to 4.5m) a total of seven length bins (each of width 0.5m) ranging from 1m to 4.5m was used to define the (assumed) multinomial distribution $p(l)$. Ideally, we would have enough length samples to estimate a time and location-specific prior age distribution, given different age-classes appear to congregate at the various sites from where genetic samples were taken. However, this was not possible given the small numbers of animals, so instead we estimate an aggregate age prior across sampling locations and time-frames.

We already have the distribution $p(l \mid a)$, so we use maximum likelihood to estimate the mean and variance of $p(a)$. A discretized lognormal distribution was assumed to be a plausible model for the prior given both the slightly right-skewed shape of the observed length distribution in the dataset and the quasi-linear nature of the length-at-age relationship over the observed length range. To be clear, we use the log-normal density to create a suitably normalised discrete prior distribution across ages 0 to 11.

S2.4.3: Estimating the posterior distribution of age given length

Once the prior age distribution was estimated, the posterior age-at-length matrix (for the 7 length bins and ages 0 to 11) was calculated using Bayes' rule. This age-at-length distribution was then combined with the year-of-capture y to calculate the cohort-at-length distribution $p(c \mid l, y)$ in the CKMR likelihood as defined in the main text.

S2.5: Dealing with intra-cohort comparisons in CKMR

One complication in HSP-based CKMR concerns intra-cohort comparisons. If there is variation between litters in number of surviving offspring— due, say, to variations in the initial litter size, or in early-life-history survival rates per litter— then there will be a systematic excess of intra-cohort sibs relative to cross-cohort sibs. The CKMR probabilities are designed to eliminate random (as opposed to systematic) variations in reproductive output, but this does not hold for intra-cohort comparisons, as explained in S2.8.1 and section 3.9 of [21]. Ignoring this phemonenon, i.e. interpreting the likely excess of intra-cohort sibs in the same way as the cross-cohort sibs, could lead to negative bias in the abundance estimates. In teleost fish, where litter sizes are large, the effects can be very large and are the main reason why effective population size, which is directly affected by intra-cohort variability, can be so much smaller than census population size in such species. For less-fecund animals such as white sharks, intra-cohort effects seem likely to be less dramatic (S2.8.1), but cannot be ruled out a priori when litter size exceeds one.

In general, the cleanest way to deal with intra-cohort sib comparisons in CKMR would be to exclude them altogether from the likelihood. However, this is not possible with our dataset since we do not know most of the birth-cohorts exactly.

The implications of intra-cohort sibship for modelling are quite subtle. Maternal and paternal effects operate differently: litter-mates are bound to share the same mother, but may or may not share the same father since multiple paternity within litters is a documented phenomenon for white sharks[7]. However, males mating with several females in the same year may contribute intra-cohort half-sibs that are *not* susceptible to shared litter-effects. And litter-mates that do share a father as well as a mother will "present" as intra-cohort FSPs not HSPs, whereas cross-cohort FSPs will be rare in random-mating species with large population sizes. To fully capture all these phenomena in CKMR would require a somewhat elaborate model and rather more kin-pairs than we have available so far.

From a more empirical perspective: based on lengths and capture-dates, about 5% of our comparisons are likely to be intra-cohort. In the absence of any litter effect (i.e. if all juveniles were produced independently in random matings and with uncorrelated survival chances), we might therefore expect to find that 1–2 of our 20–24 total HSPs–FSPs are likely intra-cohort (based on lengths etc). In fact, we found 5 such (4 FSPs and 1 HSP, which was probably maternal, based on mtDNA). While not statistically significant, this is a little higher than expected, so there could be some litter effect in white sharks. In this particular case, simply removing the FSPs (i.e. removing one animal from each FSP) happens to bring the number of likely-intra-cohort sib-pairs roughly in line with the cross-cohort sib-pairs, without needing to introduce any litter-effect complications into the CKMR model. This seems a reasonable pragmatic solution for our particular small dataset, but it could not be relied on in general. In future, using mtDNA data, we intend to construct a sex-based model allowing for multiple paternity where full-siblings, maternal half-sibs, and paternal half-sibs can be dealt with explicitly, and the litter effect directly estimated.

S2.6: Priors for life-history parameters

The juvenile survival estimates basically allow us to extrapolate back from annual adult recruitment (estimated from CKMR data) down to the youngest age of acoustically-tagged animals, i.e. 1 year olds. Survival rates for animals younger than that may well be lower, and cannot be estimated directly from our data. In order to complete the extrapolation back to the 0-group we require priors on on fecundity and year-0 survival.

S2.6.1: Annual effective fecundity

Definitive information on fecundity of female white sharks is scarce, although information does exist pertaining to litter sizes[7]. We separate the effective annual fecundity into two sub-processes: average litter size $\tilde{\alpha}$, and the mean number of years between litters y_{int}. Generally, litter sizes can be as high as 10, and can range as low as 2, though for our prior construction we worked with a range of 4–10 pups per female per reproductive event. Breeding frequency is thought to be 2 or 3 years[7]. So for the effective number of *female* pups per female per year we considered the following formulation:

$$\alpha = 0.5\tilde{\alpha}y_{\text{int}}^{-1},$$

with the factor of 0.5 reflecting the observed equal sex ratio in the pups[7]. By assuming uniform (discrete) distributions for both $\tilde{\alpha}$ and y_{int} over the already defined ranges we obtain a prior for α with median (and 95% probability interval) of 1.5 (1–2.5).

S2.6.2: Age-0 survival multiplier

For this parameter, there are no direct observations from which we can formulate a prior. However, we can reasonably expect that 0-year-old survival is very likely to be lower than for the next few age-classes. We used a beta-distributed prior for this parameter: $\delta \sim B(8, 2)$. This gave a prior median (and 95% probability interval) of 0.82 (0.52–0.97), which gave a range of neonate survival at anywhere between 50–100% of their older counterparts.

S2.6.3: Maturity-at-age relationship

To parameterise both the survival probability-at-age and the Leslie matrix for the total population estimation section, we needed to define the female maturity-at-age relationship, m_a. We used a logistic formulation:

$$m_a = \frac{1}{1 + 19\exp\left((a_{50} - a)/(a_{95} - a_{50})\right)},$$

where $a_{50} = 13$ and $a_{95} = 16$ are the assumed ages at 50% and 95% maturity, respectively[7]. The use of the number 19 in the logistic function is not specific to the white shark, but a consequence of the specific parameterisation of the logistic function. At $a = a_{50}$, we have that $m_a = (1 + 19^0)^{-1} = 0.5$; at $a = a_{95}$, we have that $m_a = (1 + 19^{-1})^{-1} = 0.95$.

S2.7: Constructing a full population estimate of abundance

We have estimates of juvenile survival (covering a range of ages from about 1–6 years old) from the acoustic tag mark-recapture analysis, and adult abundance and survival from the close-kin mark-recapture analysis. We are interested in constructing a full-population estimate of abundance because, while we are clearly

interested in the adult population from the conservation perspective, the human interaction side of the issue occurs far more frequently with younger, immature animals. Having an understanding of the whole population, both juvenile and adult, is clearly advantageous from a number of perspectives. We need to able to parameterize four key processes/variables to construct a full population abundance estimate: (i) survival probability across the population age-classes, (ii) relative fecundity-at-age, (iii) population rate of change, and (iv) *some* estimate of abundance for a knowable fraction of the population age-classes.

S2.7.1: Survival probability-at-age

We have the distributions of the juvenile (via the acoustic analysis) and adult (via the close-kin analysis) survival probabilities. What we need to do is to define some way of linking these across the age classes in the population. For ages 1 and above we define the following age-specific survival probability:

$$\phi_a = (1 - m_a)\phi^J + m_a\phi^A$$

where m_a is the maturity ogive defined in Section S2.6.3. The main idea behind this kind of transition, as opposed to a linear transition as often assumed for teleosts, is the behavioural shifts that occur as white sharks mature. In smaller teleost fish that are often prey species, survival rate often increases with length as animals "escape" the length classes that leave them vulnerable to various predators. With white sharks, their exposure to sources of mortality is driven more by where they are and what they are doing than anything explicitly size-driven like predation. As the animals begin to really ascend the maturity curve (above 4m) their spatio-temporal behaviour is fundamentally different to the juveniles[7] - they are in different places doing (and eating) different things. Obviously, we cannot be sure the transition will occur *exactly* in this manner, but we do feel it is a more realistic transition than a simple linear one. We do, however, explore the implications of alternative structural assumptions in relation to our estimates of abundance in the next section. The neonate (age 0) or young-of-the-year survival probability is assumed to be the same as age 1 *but* with the multiplier, δ, defined in Section S2.6.2 now included: $\phi_0 = \delta\phi_1$.

Although this formulation means that survival rates should increase slightly through juvenile life, in practice the changes are very small up to say age 12, an age which a very small fraction of our acoustically-tagged animals could possibly have reached during the study. Thus this formulation is compatible with the simple constant-survival CJS model (see Methods).

S2.7.2: Fecundity-at-age

We need to be able to characterise the expected number of new female offspring per-female per-year, f_a, and we do this by combining the maturity ogive, m_a, with the annual effective fecundity parameter, α, defined in Section S2.6.1 so that $f_a = \alpha m_a$.

S2.7.3: Population rate-of-change

For the close-kin analyses we assumed five scenarios encompassing various growth/equilibrium/decline scenarios: $\lambda \in \{0, \pm 0.02, \pm 0.04\}$. This choice of values was informed by both the close-kin analyses and the life-history. Figure S4 shows the HSP likelihood profile for λ in the close-kin analyses, where the maximum likelihood estimate is close to zero (-0.008), with a 90%CI of $[-0.07, 0.08]$. We are not proposing to use this distribution directly, but it does show us that the close-kin data are, at present, not very informative for this parameter. This is to be expected given the small number of HSPs found so far, and the time-coverage

of the data, relative to life-history time-scales for this species. Little to no change (15% decrease in the last 20 years) seems to be the most likely estimate, but the confidence intervals are very wide. Growth rates above 0.04 seem hard to believe given the life-history parameters we already have and use in this paper - one would not expect to see a maximum growth rate (the so-called intrinsic rate of increase r calculated from the Leslie matrix for the most optimistic life-history parameters) above 0.06, and that only by assuming no additional mortality of sharks in fishing and protection practices. Equally, rates of change below -0.04 suggest hard-to-credit current population sizes for adults that would likely be visible as bottleneck effects in the genetics, which have not been seen. So, we chose the $\lambda \in \{0, \pm 0.02, \pm 0.04\}$ range accordingly, and again we discuss the potential impact of this assumption in the following section.

S2.7.4: Abundance estimate for a subset of the population

This is the final piece of the full population abundance puzzle we require, and obviously we are going to use the close-kin estimates of adult abundance in year t, N_t^A. We assume that the maturity ogive will suffice in defining the proportion of the total population that is adult: $N_t^A = \sum_a N_{t,a} m_a$ where $N_{t,a}$ is the number of adults aged a in year t. We assume the same maturity schedule applies to males.

S2.7.5: Using Approximate Bayesian Computation to integrate the analyses

We have, so far, defined what we need to combine all the elements into the total abundance estimate, but not exactly how we can do it. Approximate Bayesian Computation, or ABC, is a powerful inference method when strict likelihoods cannot be defined[60]. Classical likelihood driven inference posits a likelihood function for the model parameters, $\boldsymbol{\theta}$, given the data, D: $\ell(D \mid \boldsymbol{\theta})$. With a prior distribution for the parameters, $p(\boldsymbol{\theta})$, we can then define the *posterior* distribution of the parameters conditional on the data via Bayes' theorem:

$$\mathbb{P}(\boldsymbol{\theta} \mid D) = \frac{\ell(D \mid \boldsymbol{\theta}) p(\boldsymbol{\theta})}{\ell(D)},$$

where $\ell(D) = \int \ell(D \mid \boldsymbol{\theta}) p(\boldsymbol{\theta}) \mathrm{d}\boldsymbol{\theta}$. The ABC approach comes into its own when we cannot really construct (or calculate) $\ell(D \mid \boldsymbol{\theta})$, and in our case it is the construction issue we face. We do not have enough informative data from across both time, t, and the age range, a, of sharks from which to accurately estimate the abundance matrix $N_{t,a}$. What we do have is posterior estimates of key parameters (juvenile survival, ϕ^J, and adult survival ϕ^A and abundance N_t^A for a given set of population growth rates, λ). These data-driven posterior estimates can be used as priors, alongside the priors for the other key life-history parameters and the range of imposed population growth rates, to estimate approximate distributions at a given time for the total abundance, N_t.

In the ABC paradigm, we relax the "likelihood given the data" requirement to one where we can simulate a particular (and hopefully informative) statistic D' from the model (defined by $\boldsymbol{\theta}$) and define a suitable distance metric $\rho(D, D')$. For a given tolerance level, $\varepsilon > 0$, if $\rho(D, D') \leq \varepsilon$ then we adjudge the given parameter vector $\boldsymbol{\theta}$ as acceptable, and not if otherwise. From this idea, we can define a very simple accept/reject algorithm to draw samples of $\boldsymbol{\theta}$ from an *approximation* to the posterior distribution $\mathbb{P}(\boldsymbol{\theta} \mid D)$.

The algorithm[60] proceeds as follows:

1. Generate a proposal $\boldsymbol{\theta}^*$ from the prior $p(\boldsymbol{\theta})$
2. Simulate D'^* from the model defined by $\boldsymbol{\theta}^*$

3. Calculate the distance $\rho(D, D'^*)$

4. If $\rho(D, D'^*) \leq \varepsilon$ accept $\boldsymbol{\theta}^*$; if not, return to first step

By repeating the above algorithm until we have obtained a suitable number, we have generated our sample of $\boldsymbol{\theta}$ from the approximate posterior of interest.

For the white shark application D is in fact the logarithmic population growth rate, λ. Obviously, we are not in a position to define a likelihood for this variable given the close-kin data are uninformative in this regard. We have, however, formed a plausible list of 5 alternatives: $\lambda = \{0, \pm 0.02, \pm 0.04\}$. Our parameter vector of interest is $\boldsymbol{\theta} = \{\alpha, \delta, \phi^J, \phi^A\}$ with the priors for α and δ as defined in Sections S2.6.1 and S2.6.2 respectively, and for ϕ^J and ϕ^A from the acoustic and close-kin mark-recapture analyses respectively. Together

The simulated value of λ is derived using a Leslie matrix model for the total population: $N_{t+1,a+1} = \boldsymbol{\Lambda} N_{t,a}$. The first row of $\boldsymbol{\Lambda}$ is defined by the fecundity-at-age $f_a = \alpha m_a$. The $\{a+1, a\}$-th entry of $\boldsymbol{\Lambda}$ is given by the survival probability ϕ_a. We choose a maximum age of 60, so for the average adult survival probability to be comparable to the close-kin estimate (where no maximum age is assumed) we make a minor adjustment (depending on λ and age-at-maturity) to the nominal prior survival probability before it is used in the ABC algorithm. The model-predicted estimate of λ, $\widehat{\lambda}$, is the logarithm of the lead Eigenvalue of $\boldsymbol{\Lambda}$, with the associated Eigenvector being the stable age distribution, ζ_a. The tolerance factor $\varepsilon = 0.005$, so if $|\lambda - \widehat{\lambda}| < 0.005$ we accept the proposed quarter $\boldsymbol{\theta} = \{\alpha, \delta, \phi^J, \phi^A\}$. To get total abundance we make use of the stable age distribution, ζ_a. Any given sample of ϕ^A has an associated value of the adult abundance, N_t^A, and we take $t = 2015$ for the paper calculations on total abundance. If total abundance is given by N_t, and we assume maturity partitions the adult population, then the following equation holds true:

$$N_t^A = N_t \sum_a m_a \zeta_a,$$

where $\sum_a \zeta_a = 1$, and so

$$N_t = \frac{N_t^A}{\sum_a m_a \zeta_a}.$$

We calculated N_t for each of the 1,000 samples from the approximate parameter posterior distribution and for each of the 5 population rate-of-change scenarios. Table 2 in the main text details the full posterior summaries for the parameters in the ABC algorithm: $\boldsymbol{\theta} = \{\alpha, \delta, \phi^J, \phi^A\}$ and for all the population rate-of-change scenarios. Note that this population dynamics model does not directly require parameters for male fecundity-at-age. However, fathers do of course contribute half of the HSPs, so it is still necessary to include their dynamics, and to define the proportion of males that are adult, and to interpolate juvenile to adult survival rates for males. We have assumed a 50/50 sex ratio throughout lifespan (which is consistent with data from neonates and juveniles), and that the maturity schedule for females applies to males too.

S2.8: Implications of key structural uncertainties

Our approach attempts to capture all main sources of quantitative uncertainty arising from limited data and prior information. One thing that will always remain, though, isstructural uncertainty: key assumptions we have to make and where, although alternatives might exist, we cannot statistically choose between them. In this section, we attempt to at least address the potential implications of some structural uncertainties on the final abundance estimates.

S2.8.1: No age-effects in close-kin analyses

We assume that all adults of given sex are equal in reproductive terms: hence the lack of any explicit age- or size-structure after maturity in the close-kin mark-recapture model. While this seems defensible for white sharks, as explained below, we should elaborate on what this might mean if it is incorrect.

Note that there are three aspects to consider: (i) whether all adults of given size (and sex) have similar fecundity (i.e. expected reproductive output of surviving juveniles), (ii) whether there is a body-size (or age) effect on average fecundity, and (iii) whether there is likely to be substantial within-lifespan change in fecundity for each individual post maturity. These need not hold in general. In teleost fish, for example, (i) is plausible but both (ii) and (iii) are usually not: body size does affect fecundity, individuals grow substantially after maturity, and individuals vary in size-at-age. In territorial mammals, for example, (ii) and (iii) might hold but (i) may not if some territories are more productive. However, white sharks are not territorial, show limited growth after maturity, and do not exhibit any clear relationship between body-size and fecundity (at least for females; males have not been studied), so our assumption seems reasonable. Nevertheless, it is instructive to consider what the implications might be if not. Here we focus on within-life changes in fecundity— item (iii)— arising from the non-step-change transition to maturity in S2.7.3. Because the transition takes only a small proportion of the expected life-span of adults, the effect ought intuitively to be small, but is worth checking.

The first task is to generalize the formula for the probability of HSPs, using the principles explained in section 3.9 of [21]. Suppose we are comparing two juveniles i and j of known age born in cohorts (years) c_i and c_j, where i was born first so that $c_i < c_j$ (strict inequality, i.e. different cohorts). Consider all potential mothers of i, i.e. all females alive at c_i. The probability that one particular female aged a at that time is i's mother, is her Expected Relative Reproductive Output (ERRO, as per reference[21]) in year c_i: in other words, her likely output as a proportion of the total reproductive output of all females that year. Symbolically, her ERRO is

$$\frac{f_a}{\sum_{a'} f_{a'} N_{\female c_i, a'}}$$

where f_a is female fecundity-at-age and $N_{\female}$ is female abundance. For the same female to also be j's mother, she must first of all survive until c_j; if she does, her fecundity (i.e. average reproductive output) will have changed. Her probability of being j's mother (conditional on being alive) is her ERRO in c_j, relative to the total from all females alive in c_j. Putting this together algebraically, and summing across all the females alive in c_i taking into account numbers-at-age, yields the following:

$$\mathbb{P}\left[i \text{ and } j \text{ have same mother} | c_i, c_j\right] = \sum_a N_{\female c_i, a} \left(\frac{f_a}{\sum_{a'} f_{a'} N_{\female c_i, a'}} \times \prod_{b=a}^{a+\delta_{ij}-1} \phi_b \times \frac{f_{a+\delta_{ij}}}{\sum_{a''} f_{a''} N_{\female c_j, a''}} \right)$$

where ϕ_b is the annual survival probability at age b, and $\delta_{ij} = c_j - c_i$. If fecundity is equal for all mature females, and survival-at-age is constant throughout adulthood at value ϕ, several terms cancel and the formula reduces to

$$\mathbb{P}\left[i \text{ and } j \text{ have same mother} | c_i, c_j\right] = \frac{\phi^{c_j - c_i}}{N^A_{\female c_j}}$$

from which formula (4) in Methods follows directly, by assuming a 50-50 sex ratio and that males too are reproductively equal as adults.

Note that this deliberately ignores skip-spawning, although it is technically incorrect to do so. If females breed on a rigid two-year schedule, for example, the probability should actually be zero if $c_j - c_i$ is an odd number of years. However, only half the female population will be breeding in any given year, so if $c_i - c_j$ is even then the probability should be twice as high as written. These factors basically cancel out when studying a long-lived species using a wide range of juvenile cohorts.

Note also that the derivation illustrates the difficulty with intra-cohort comparisons, when $c_i = c_j$. In that case, it is not the *expected* reproductive output of the mother that matters, but her *actual* output instead. It appears as a squared term, so random reproductive variability as well as the expected value becomes relevant. When $c_i \neq c_j$, though, two independent random events are being considered, and the expected values can simply be multiplied. The key point is that the individual's reproductive output in the two years should be statistically independent conditional on the individual covariates included in the model— i.e. whether the potential parent is still alive, plus any covariates that have persistent effects on its fecundity (in this case, age).

It is hard to second guess the general influence of age structure, so a simple but relevant example may help here. For a "white shark like" example, and removing the effect of age-structured survival probability for ease of interpretation, assume that $f_a \equiv m_a$, and $\phi_a \equiv 0.95$. To simulate the time difference between cohorts that looks something like that in our study, we assume a gamma distribution for δ_{ij} with shape and rate parameters 1.24 and 0.21, respectively. Since the explicit survival term is the same as for the age-aggregated model, and the age distribution is assumed stable, we are only concerned with the values of the following:

$$\xi_{ij} = \frac{\sum_a \left(f_a N_{♀a} f_{a+\delta_{ij}}\right)}{\sum_{a'} \left(f_{a'} N_{♀,a'}\right)}$$

Given our assumed distribution for δ_{ij}, the median (and 95% range) for ξ_{ij} was 0.99 (0.95–0.995), with lower values of ξ_{ij} associated with smaller values of δ_{ij}. So, at least for the simplified example detailed here, there is the possibility of half-sibling probability being very slightly over-estimated, and abundance under-estimated, by ignoring age-structure. This potential for bias seems to be reduced with higher survival probabilities and wider range of cohorts in the dataset.

S2.8.2: Using maturity to transition from juvenile to adult survival

As already stated, we cannot truly know how survival changes in between the acoustic data (ages 1–6) and close-kin data (generally ages 13 and above) ranges. We argued for a transition in line with the maturation schedule as opposed to a linear transition for what we think are sensible reasons (see Section S2.7.1) but what if the transition occurred faster than we assumed? The key anchoring factor for total abundance is the close-kin estimate, N_t^A, but survival dictates how "fast" we get there in terms of the juvenile dynamics. If we take the age-at-50% maturity, a_{50} as a useful reference age, then our assumptions about how the survival probability transition occurs will - all other things being equal - fix the probability of surviving *to* age a_{50}:

$$\nu_{a_{50}} = \prod_{a=0}^{a_{50}-1} \phi_a.$$

If that transition happens faster than the maturation schedule (e.g. linearly) then, for the same overall population growth rate, $\nu_{a_{50}}$ will increase but N_t^A has not changed. If we assume to hold survival fixed, then effective fecundity and overall abundance *must* decrease to obtain the pre-set adult abundance level. The opposite would happen if the transition to adult survival was slower than the maturation schedule and total abundance would increase. This is not exactly analogous to our case, since we allow the estimation of both juvenile and adult survival (albeit with strongly informative priors) as well as effective fecundity and the 0-group survival multiplier, but something similar would presumably occur. The general conclusion is this: if the transition from juvenile to adult survival is faster/slower than we modelled it to be (via maturity) then we are probably under/over estimating the total population abundance.

References

43. Courtois, B *et al.* Genome-wide association mapping of root traits in a japonica rice panel. *Plos One* **8**, e78037 (2013).

44. Cruz, V. M. V, Kilian, A. & Dierig, D. A. Development of DArT marker platforms and genetic diversity assessment of the US collection of the new oilseed crop lesquerella and related species. *Plos One* **8**, e64062 (2014).

45. Kilian, A *et al.* "Diversity arrays technology: a generic genome profiling technology on open platforms" in Data Production and Analysis in Population Genomics. (Springer, New York, 2012), pp. 67–89.

46. Raman, H. *et al.* Genome-wide delineation of natural variation for pod shatter resistance in Brassica napus. *Plos One* **9**, e101673 (2014).

47. Baird, N. A. *et al.* Rapid SNP discovery and genetic mapping using sequenced RAD markers. *Plos One* **3**, e3376 (2008).

48. Elshire, J. R. *et al.* A robust, simple genotyping-by-sequencing (GBS) approach for high diversity species. *Plos One* **6**, e19379 (2011).

49. Grewe, P. G. *et al.* Mitochondrial DNA variation among lake trout (*Salvelinus namaycush*) strains stocked into Lake Ontario. *Can. J. Fish. Aquat. Sci* **50**, 2397–2403 (1993).

50. Chang, C. H., Shao, K.T., Lin, Y. S., Fang, Y. C., & Ho, H. C. The complete mitochondrial genome of the great white shark, *Carcharodon carcharias* (Chondrichthyes, Lamnidae). *Mitochondrial DNA* **25**, 357–358 (2014).

51. Feutry, P. *et al.* Mitogenomics of the Speartooth Shark: challenges after ten years of control region sequencing. *BMC Evol. Biol.* **14**, 232 (2014).

52. Chen, X., Liu, M., Grewe, P. G., Kyne, P. M., & Feutry, P. Complete mitochondrial genome of the Critically Endangered speartooth shark *Glyphis glyphis* (Carcharhiniformes: Carcharhinidae). *Mitochondrial DNA* **25**, 431–432 (2014).

53. Li, H. *et al.* The Sequence Alignment/Map format and SAMtools. *Bioinformatics* **15**, 2078–2079 (2009).

54. Kery, M., & Schaub, M. *Bayesian population analysis using WinBUGS: a hierarchical perspective.* (Academic Press, 2011).

55. Carpenter, B. *et al.* Stan: A probabilistic programming language. *J. Stat. Soft.* **20**, 1–37 (2016).

56. Gelman, A., Carlin, J. B., Stern, H. S., & Rubin, D. B. *Bayesian data analysis* (Chapman and Hall,

New York, ed. 2, 2006).

57. Butler, K., & Stephens, M. *The distribution of a sum of binomial random variables* (Tech. Rep. 467, Office of Naval Research. 1993).

58. Anderson, E. C. & Garza, J. C. The power of single-nucleotide polymorphisms for large-scale parentage inference. *Genetics* **172**, 2567–2582 (2006).

59. Hillary, R. M. Practical uses of non-parametric methods in fisheries assessment modelling. *Mar. Fresh. Res.* **63**, 606–615 (2012).

60. Marjoram, P., Molitor, J., Plagnol, V. & Tavaré, S. Markov chain Monte Carlo without likelihoods. *Proc. Natl. Acad. Sci.* **100**, 15,324–15,328 (2003).

Figures

Fig. S1

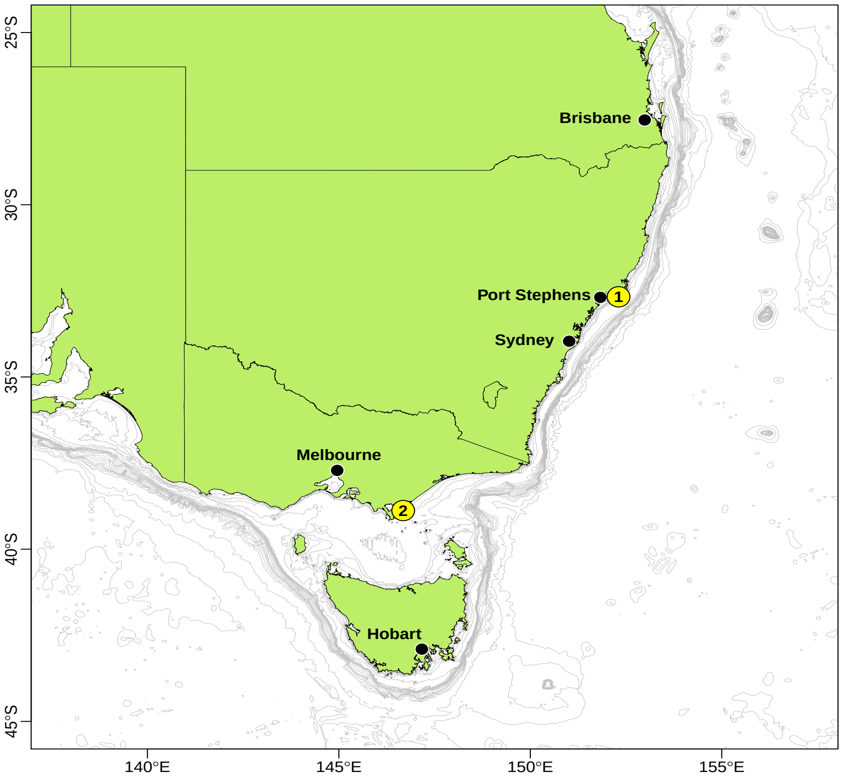

Locations of tag release along the coastlines of New South Wales (location 1) and South East Victoria (location 2), along the East Coast of Australia. The map figure was generated using the latest versions of the R[39] packages `ggplot2`[40], `ggmap`[41], and `gridExtra`[42], available on the Comprehensive R Archive Network `https://cran.r-project.org/`.

Fig. S2

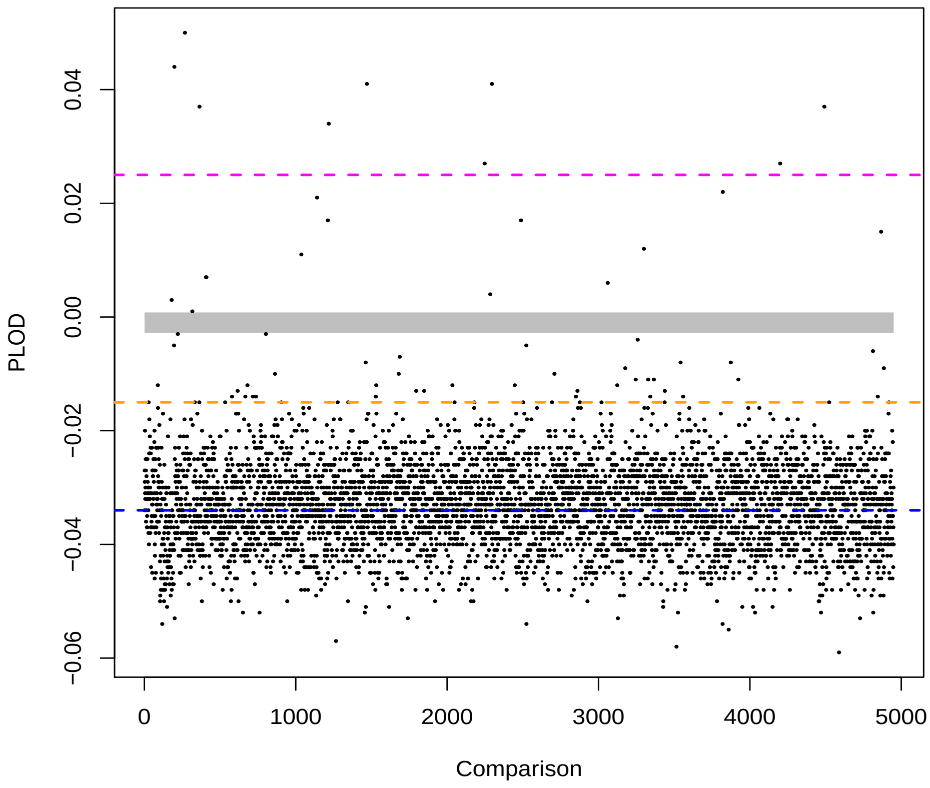

$\text{PLOD}_{\text{up}}^{\text{hsp}}$ for the 97 Eastern fish without FSPs. The blue, orange and magenta dotted lines denote the expected values for unrelated, first cousins, and half-siblings, respectively. The bottom of the grey shaded region denotes the false positive cut-off for C1Ps; the top denotes the PLOD level below which we would expect to see less than 1 true HSP.

Fig. S3

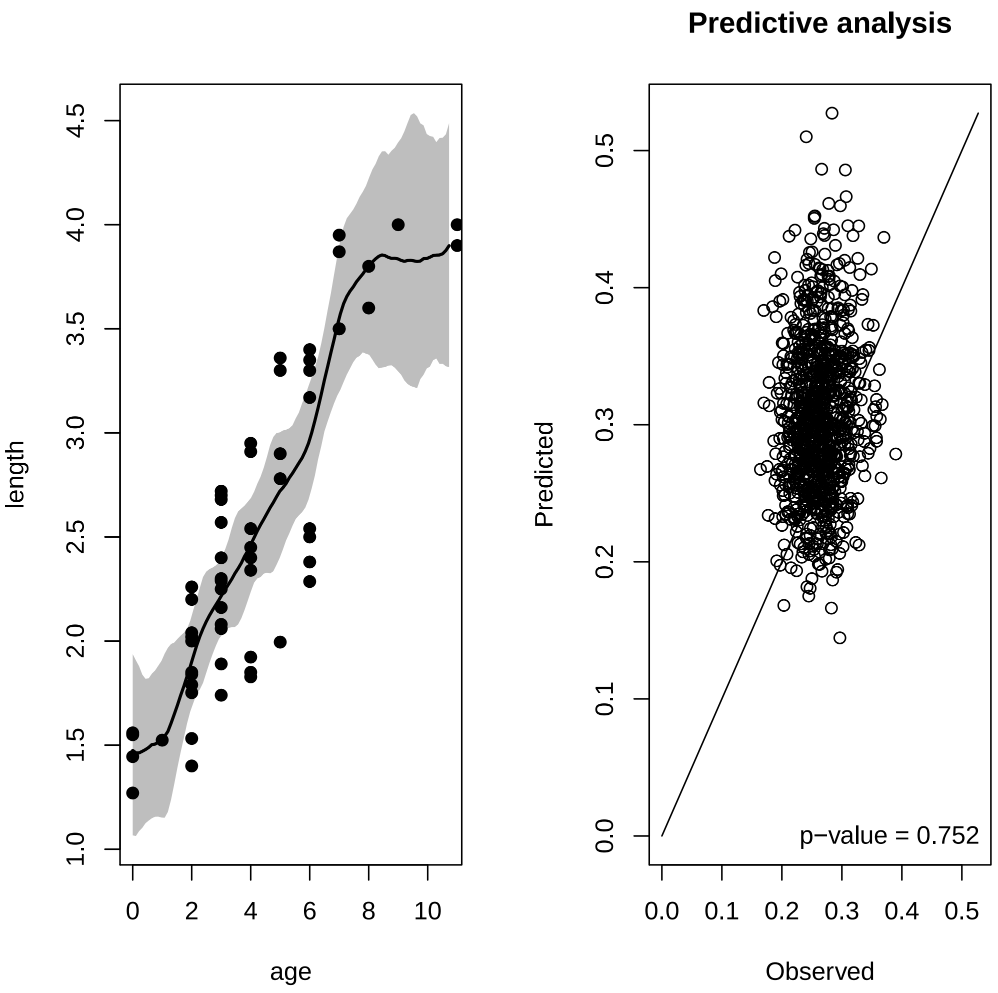

On the left is the observed length-at-age (black dots) and the posterior median (black line) and 95% credible interval (grey shading) expected length-at-age. On the right is the distribution of the posterior predictive discrepancy statistics and associated p-value of 0.752.

Fig. S4

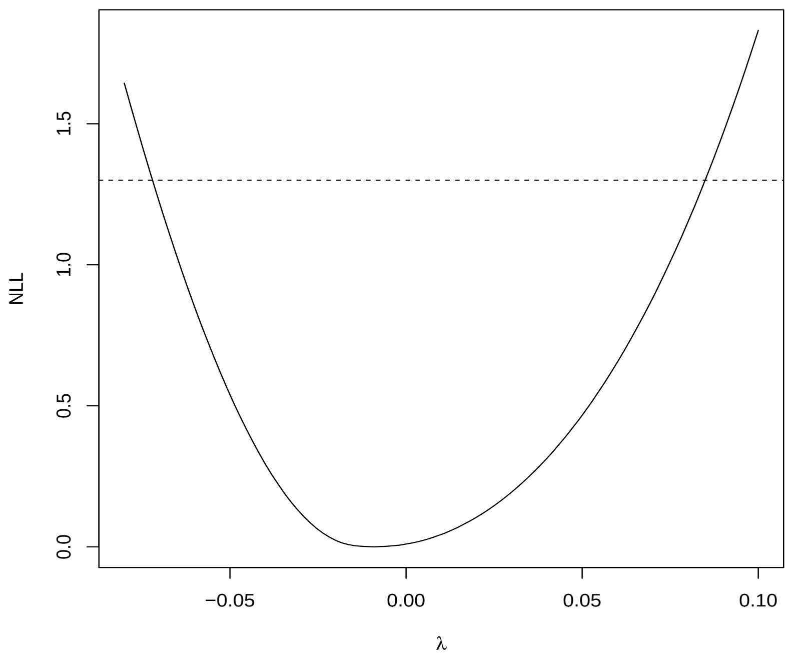

Likelihood profile for the population rate-of-change parameter, λ, from the close-kin analyses. The negative log-likelihood (NLL) is normalised to have a minium of zero and the interval between where the black dotted line intersects the solid curve is the approximate 90%CI for λ (-0.07 to 0.08).

Table S1

	**A** (8231bp)
WS(9964ArgL-For)	5'-GCGCAGAATAGATACTTAGTCCAAAACAAAGAC
16sbr-H-Rev	5'-CCGGTCTGAACTCAGATCACGT
	**B** (9263bp)
WS(VAL1053-For)	5'-TCCCTTACACCGAGGAAATACTCGTGCAAT
WS'ND4L287-Rev	5'-GTAAATTATCGGAACCATGTGAGCGTGA

Primer pairs used to amplify white shark mtDNA amplicons A and B.

Table S2

Tag model	Sex	Tagging area	n (deployed)
v16-6x	f	SE Victoria	1
v16-6x	m	SE Victoria	3
v16-6h	f	Hawks Nest, NSW	7
v16-6x	f	Hawks Nest, NSW	13
v16-6h	m	Hawks Nest, NSW	1
v16-6x	m	Hawks Nest, NSW	8
v16-6h	f	Stockton, NSW	6
v16-6x	f	Stockton, NSW	3
v16-6h	m	Stockton, NSW	4
v16-6x	m	Stockton, NSW	3

Tag release details showing vemco tag model number, sex of tagged shark and area. See Fig. S1 for a map of the tagging release areas.

Table S3

Sex	Year	N(deployed)
f	2007	6
m	2007	4
f	2008	6
m	2008	1
f	2009	5
m	2009	3
f	2010	4
m	2010	3
f	2011	5
m	2011	3
f	2012	4
m	2012	1
f	2013	0
m	2013	3
f	2014	0
m	2014	1

Numbers of acoustic tags deployed by year and sex.

Table S4

	2008	2009	2010	2011	2012	2013	2014
T08.32	-	1.00	1.00	1.00	0.00	0.00	0.00
T08.33	-	1.00	1.00	1.00	0.00	0.00	0.00
T08.34	1.00	1.00	1.00	1.00	0.00	0.00	0.00
T08.35	-	1.00	1.00	1.00	0.00	0.00	0.00
T08.36	-	1.00	1.00	0.00	0.00	0.00	0.00
T08.37	-	1.00	0.00	0.00	0.00	0.00	0.00
T08.38	-	1.00	0.00	0.00	0.00	0.00	0.00
T09.01	-	1.00	1.00	1.00	1.00	1.00	0.00
T09.02	-	-	1.00	1.00	1.00	1.00	1.00
T09.18	-	-	1.00	0.00	0.00	0.00	0.00
T09.19	-	-	1.00	0.00	0.00	0.00	0.00
T09.20	-	-	1.00	0.00	0.00	0.00	0.00
T09.21	-	1.00	1.00	1.00	0.00	0.00	0.00
T09.22	-	-	1.00	1.00	0.00	0.00	0.00
T09.23	-	-	1.00	1.00	1.00	0.00	1.00
T10.25	-	-	1.00	1.00	1.00	0.00	0.00
T10.26	-	-	1.00	1.00	1.00	1.00	1.00
T10.27	-	-	-	1.00	0.00	0.00	0.00
T10.28	-	-	1.00	1.00	0.00	0.00	0.00
T10.29	-	-	-	1.00	1.00	1.00	1.00
T10.30	-	-	1.00	0.00	0.00	0.00	0.00
T11.01	-	-	-	1.00	1.00	1.00	0.00
T11.02	-	-	-	1.00	1.00	1.00	0.00
T11.03	-	-	-	1.00	1.00	1.00	1.00
T11.04	-	-	-	1.00	1.00	1.00	1.00
T11.10	-	-	-	1.00	0.00	0.00	0.00
T11.11	-	-	-	1.00	0.00	1.00	0.00
T11.13	-	-	-	1.00	0.00	0.00	0.00
T11.14	-	-	-	1.00	1.00	1.00	0.00
T12.01	-	-	-	-	-	1.00	0.00
T12.02	-	-	-	-	1.00	1.00	1.00
T12.03	-	-	1.00	0.00	1.00	1.00	1.00
T12.04	-	-	-	-	1.00	1.00	0.00
T12.05	-	-	-	-	1.00	1.00	0.00
T13.09	-	-	-	-	-	1.00	1.00
T13.10	-	-	-	-	-	-	1.00
T14.01	-	-	-	-	-	1.00	1.00

Capture histories for tagged sharks. The left-most column denotes shark IDs. Column headers show the year of release, and with each shark the year of release relates to the first number "1" in the capture history, with all subsequent zeroes relating to years the animal was not detected on the acoustic array, and ones relating to years that it was detected.

Table S5

	Median	20%	80%
ϕ^J	0.73	0.67	0.78
p_{obs}	0.89	0.84	0.93

Median and 80% credible intervals on juvenile survival ($\hat{\phi}^J$) and observation (recapture) probability ($\hat{p}_{obs}$).
